# Evaluation of the PPAR-γ Agonist Pioglitazone in Mild Asthma: A Double-Blind Randomized Controlled Trial

**DOI:** 10.1371/journal.pone.0160257

**Published:** 2016-08-25

**Authors:** J. R. Anderson, K. Mortimer, L. Pang, K. M Smith, H. Bailey, D. B. Hodgson, D. E. Shaw, A. J. Knox, T. W. Harrison

**Affiliations:** 1 Nottingham Respiratory Research Unit, University of Nottingham, Clinical Sciences Building, City Hospital, Hucknall Road, Nottingham, NG5 1PB, United Kingdom; 2 Liverpool School of Tropical Medicine, Liverpool, UK and Aintree University Hospital NHS Foundation Trust, Fazakerley, United Kingdom; Universite de Bretagne Occidentale, FRANCE

## Abstract

**Background:**

Peroxisome proliferator-activated receptor gamma (PPAR-γ) is a nuclear receptor that modulates inflammation in models of asthma. To determine whether pioglitazone improves measures of asthma control and airway inflammation, we performed a single-center randomized, double-blind, placebo-controlled, parallel-group trial.

**Methods:**

Sixty-eight participants with mild asthma were randomized to 12 weeks pioglitazone (30 mg for 4 weeks, then 45 mg for 8 weeks) or placebo. The primary outcome was the adjusted mean forced expiratory volume in one second (FEV_1_) at 12 weeks. The secondary outcomes were mean peak expiratory flow (PEF), scores on the Juniper Asthma Control Questionnaire (ACQ) and Asthma Quality of Life Questionnaire (AQLQ), fractional exhaled nitric oxide (FeNO), bronchial hyperresponsiveness (PD_20_), induced sputum counts, and sputum supernatant interferon gamma-inducible protein-10 (IP-10), vascular endothelial growth factor (VEGF), monocyte chemotactic protein-1 (MCP-1), and eosinophil cationic protein (ECP) levels. Study recruitment was closed early after considering the European Medicines Agency’s reports of a potential increased risk of bladder cancer with pioglitazone treatment. Fifty-five cases were included in the full analysis (FA) and 52 in the per-protocol (PP) analysis.

**Results:**

There was no difference in the adjusted FEV_1_ at 12 weeks (-0.014 L, 95% confidence interval [CI] -0.15 to 0.12, p = 0.84) or in any of the secondary outcomes in the FA. The PP analysis replicated the FA, with the exception of a lower evening PEF in the pioglitazone group (-21 L/min, 95% CI -39 to -4, p = 0.02).

**Conclusions:**

We found no evidence that treatment with 12 weeks of pioglitazone improved asthma control or airway inflammation in mild asthma.

**Trial Registration:**

ClinicalTrials.gov NCT01134835

## Introduction

Asthma is a common and chronic condition that affects 235 million people worldwide and is responsible for substantial healthcare expenditures [1]. Inhaled corticosteroids and bronchodilators are the mainstay of asthma pharmacotherapy. Although these treatments are highly effective, many patients fail to achieve optimal asthma control and are at risk of impaired quality of life, recurrent exacerbations, and even death. In some patients, these outcomes are due to poor concordance with medication, while others dislike or exhibit reduced responses to inhaled medications, creating a need to identify novel therapeutic targets if the burden of asthma is to be reduced.

The nuclear transcription factor peroxisome proliferator-activated receptor gamma (PPAR-γ) is an important regulator of cellular homeostasis and energy metabolism that also has anti-inflammatory properties [2–7]. There is evidence that PPAR-γ is involved in inflammation and airway remodeling responses in asthma [4]. In experimental models of asthma, the activation of PPAR-γ suppresses the increase in airway hyperresponsiveness, eosinophil influx, and expression of Th-2 cytokines that occur in the lung following an allergen challenge [5, 6, 8–10].

PPAR-γ is thought to exert its immune modulating effects by reducing the downstream gene transcription of targets of the pro-inflammatory transcription factors activator protein-1 (AP-1) and nuclear factor-kappaB (NF-kB) [11–13]. In cultured human airway smooth muscle cells (HASMs), PPAR-γ activation suppresses the release of the chemokines MCP-1 (monocyte chemotactic protein-1/CCL2) and eotaxin (CCL11) in response to the potent inflammatory stimulant tumor necrosis factor-alpha (TNF-α) [3]. This suppression of eotaxin release is secondary to reduced histone acetylation and reduced binding of NF-kB to the eotaxin promoter, reducing gene transcription [3].

The PPAR-γ agonists pioglitazone and rosiglitazone were developed to treat the hyperglycemia and insulin resistance of type 2 diabetes mellitus, and were used widely until rosiglitazone was withdrawn due to an adverse cardiovascular safety profile [14–16]. Prior to its withdrawal, rosiglitazone had been studied in two short-term (up to 28 days) randomized controlled trials (RCTs) in asthma that found modest effects on the late asthmatic reaction and airway obstruction in smokers with asthma [17, 18]. A longer term study of pioglitazone in a subset of obese subjects with poorly controlled asthma resulted in no significant improvement in measures of asthma control [19]. The aim of the present study was to test the hypothesis that the activation of PPAR-γ with pioglitazone (which has a more favorable safety profile than that of rosiglitazone) would improve measures of asthma control and markers of airway inflammation over a 12-week period in mild asthma. Study recruitment was terminated after 68 participants had been recruited after reports emerged that pioglitazone treatment may be associated with an increased risk of developing bladder cancer [20].

## Materials and Methods

### Ethics statement

The study was approved by Nottingham Research Ethics Committee 2 (08/H0408/120) and the Medicines and Healthcare Products Regulatory Agency (MHRA) (08028). All participants provided written informed consent. Trial registration: NCT01134835.

### Study design and participants

A single-center randomized, double-blind, placebo-controlled, parallel-group trial comparing pioglitazone and placebo over 12 weeks in adults with mild asthma was performed at City Hospital, Nottingham, UK. The study design was approved by Nottingham Research Ethics Committee 2 (08/H0408/120). All participants gave written informed consent. The full trial protocol is given in S1 Text.

Potential participants aged 18–75 years with a clinical diagnosis of asthma who were taking 0–800 μg of inhaled beclomethasone dipropionate (or equivalent) and short-acting β_2_-agonists (SABAs) as required were identified from primary care practices and our research database between January 2010 and February 2012. At screening, participants were required to have a post-bronchodilator forced expiratory volume in one second (FEV_1_) ≥ 60% of predicted with ≥ 12% reversibility of the pre-bronchodilator FEV_1_ with 400 μg of salbutamol or ≥ 12% peak flow variability during the run-in period (maximum peak expiratory flow [PEF] minus minimum PEF divided by average PEF) to be eligible.

The exclusion criteria included an exacerbation of asthma within six weeks, current smoking, or a history of more than 10 pack years of smoking, or treatment with leukotriene antagonists, long-acting beta-agonists, theophylline, or oral steroids. Patients with a history of diabetes or liver or cardiovascular disease; patients who were pregnant, lactating, or not using adequate contraception; or patients with contraindications to pioglitazone were excluded for safety reasons.

#### Screening visit

FEV_1_ was measured as the greater of two values within 100 ml using a dry bellows spirometer (Vitalograph, Buckingham, UK), prior to and 15 minutes after the inhalation of 400 μg of salbutamol via a spacer (Volumatic^TM^, Allen & Hanburys Ltd, Middlesex, UK). Sputum induction with hypertonic saline was performed as described previously [21, 22] using the ultrasonic nebulizer OMRON NE-U17 (OMRON Health Care Ltd, Milton Keynes, UK). A clinician reviewed and examined participants at each visit.

#### Run-in period

Participants entered a two-week run-in period, during which morning and evening PEF (Mini-Wright, Clement Clarke International Ltd, Harlow, UK) and asthma medication use were recorded.

#### Randomization

Eligible participants were randomized to 12 weeks pioglitazone (30 mg once a day for 4 weeks, then 45 mg once a day for 8 weeks) or matched placebo using an internet-based randomization schedule stratified by inhaled corticosteroid use. The allocation sequence was generated using random permuted blocks of two, four, and six and maintained on a secure server by the Nottingham Clinical Trials Unit (CTU).

The baseline clinical measurements were: the fractional exhaled nitric oxide (FeNO) in parts per billion (ppb) at 50 mL/second (NIOX® Flex, Aerocrine, Solna, Sweden), pre-bronchodilator FEV_1_, airway hyperresponsiveness to methacholine (PD_20_, Ganshorn ProvoJet, Ganshorn Medizin Electronic GmbH, Niederlauer, Germany) based on the protocol of Yan, Asthma Control Questionnaire (ACQ), Asthma Quality of Life Questionnaire (AQLQ), and induced sputum [23–25]. Study visits were at same time of day +/- 1.5 hours at 4, 8, 12, and 16 weeks within three days of the protocol date and the baseline clinical measurements were repeated after 12 weeks of treatment.

#### Study intervention

Pioglitazone tablets were over encapsulated with DBcaps® capsules (Capsugel Belgium NV, Bornem, Belgium), and a matched placebo backfilled with lactulose was manufactured by Catalent Pharma Solutions, Bolton, UK. An independent pharmacist issued 30 capsules of the study medication to each participant every four weeks and compliance was assessed by counting the returned capsules. All participants and clinical investigators performing the clinical assessments were blinded to allocation until all trial procedures were performed and the analysis was complete.

#### Outcome measures and statistical analysis

The primary outcome measure was the adjusted mean FEV_1_ at 12 weeks compared between treatment groups by analysis of covariance (ANCOVA) incorporating terms for baseline value, treatment arm, inhaled corticosteroid use, age, gender, and height. The secondary outcome measures at 12 weeks included mean morning and evening PEF for 14 days prior to the study visit, ACQ and AQLQ scores, FeNO, PD_20_, SABA use, and body mass index (BMI). We considered a PD_20_ of 800 μg to be equivalent to a PC_20_ of 8 mg/mL [26]. Final follow up visits were in December 2011.

In subsets of participants, induced sputum cell counts, sputum supernatant interferon gamma-inducible protein-10 (CXCL10/IP-10), vascular endothelial growth factor (VEGF), MCP-1/CCL2, and eosinophilic cationic protein (ECP) were measured. Activation of PPAR-γ was assessed by comparing levels of cytoplasmic and nuclear PPAR-γ from induced sputum cells between groups. Adverse effects (reported spontaneously at clinic visits and on diary cards) were recorded, and random glucose, full blood count (FBC), and liver function tests (LFTs) were measured pre- and post-treatment.

Where appropriate, secondary endpoints were log-transformed to approximate a normal distribution (PD_20_, percentage eosinophil count, and FeNO) and compared between groups using ANCOVA, incorporating terms for baseline value treatment arm, inhaled corticosteroid use, and, where appropriate, age, gender, and height. Values that could not be transformed were compared using the Mann-Whitney U test. In participants with values below the lower limit of detection in laboratory assays (five participants for ECP), a value of 0.5 of the lower detection limit was used. Sputum mediators were expressed per 1 x 10^6^ cells and were reported as geometric means with 95% confidence intervals (CIs). Stata v11.0 (StataCorp LP, College Station, TX, USA) and GraphPad Prism Version 6 (GraphPad Software, La Jolla, CA, USA) were used to perform the statistical analyses.

The *a priori* calculation suggested that 88 participants would allow us to detect a difference of 150 mL between groups in the mean FEV_1_ at 12 weeks, with 80% power at a 5% two-sided significance level, assuming a standard deviation (SD) of FEV_1_ over eight weeks of 250 mL [27].

A Trial Steering Committee (TSC) and independent Data Monitoring and Ethics Committee (DMEC) monitored the progress and safety of the trial.

#### Sputum processing

Induced sputum samples were processed within two hours of induction as described previously [21, 22]. Sputum supernatant IP-10, MCP-1, and VEGF were assayed using the Bio-Plex® 200 system (Bio-Rad, Hemel Hempstead, UK) and supernatant ECP was assayed using ImmunoCAP 100 (Phadia AB, Uppsala, Sweden) according to the manufacturers’ instructions. PPAR-γ was assayed in the nuclear and cytoplasmic fractions of sputum cells using a TransAM™ PPAR-γ Kit (Active Motif, Rixensart, Belgium) and a competitive enzyme-linked immunosorbent assay (ELISA) PPAR-γ kit (Biosources, Inc., San Diego, CA, USA) according to the manufacturers’ instructions. The optical density at 450 nm (OD_450_) was used for quantification of nuclear PPAR-γ and the reciprocal of the OD_450_ was used for quantification of cytoplasmic PPAR-γ.

## Results

**Fig 1:**
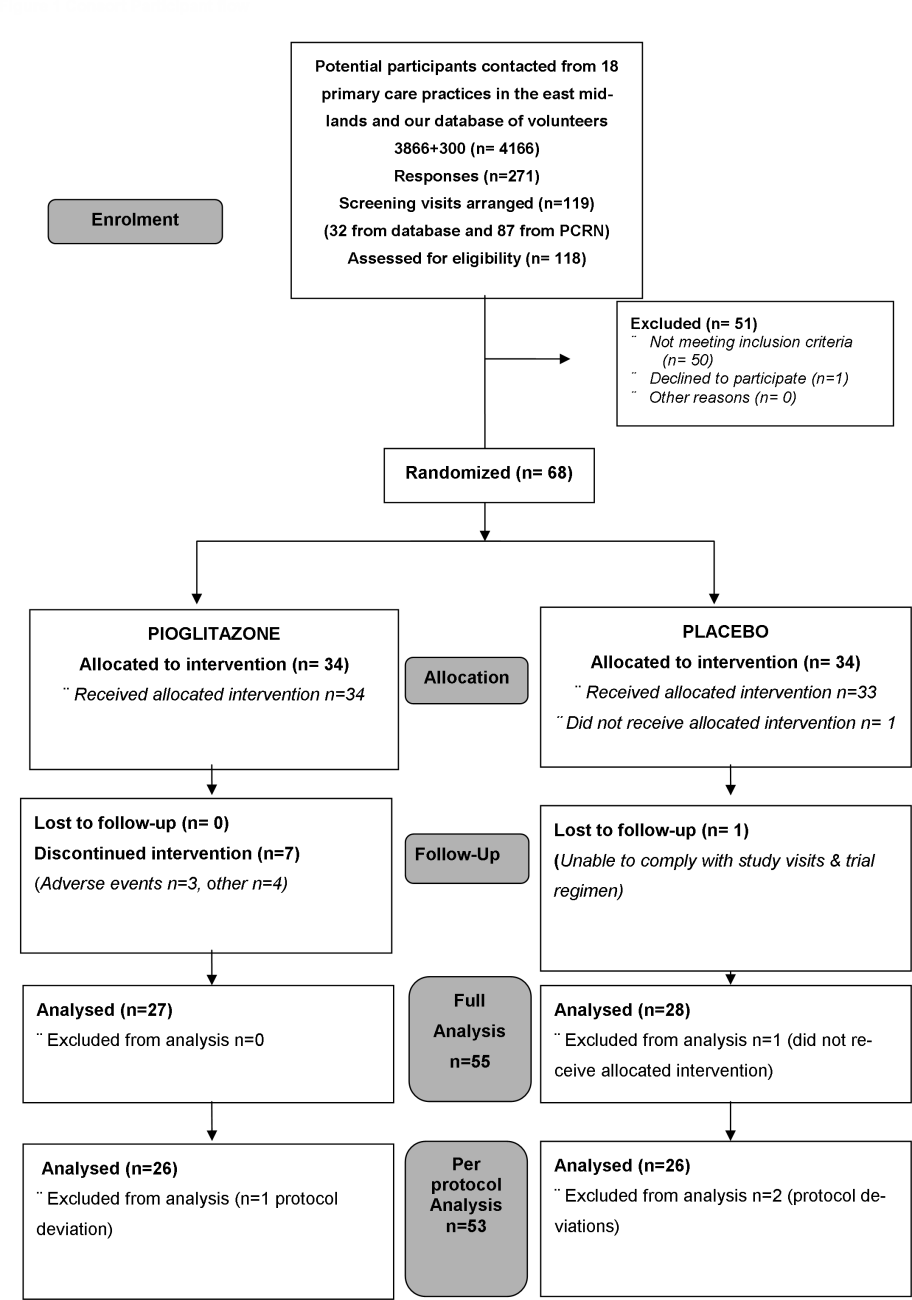
Consort participant flow.

Of the 119 individuals screened, 68 participants were randomized, 34 to pioglitazone and 34 to placebo between January 2010 and February 2012 (Fig 1). The full trial dataset is given in S1 Data and Consort checklist in S2 Text.

The full analysis was performed on 55 completed cases, and after excluding those with protocol deviations, the per-protocol analysis included 52 participants. The baseline characteristics of the study population are shown in Table 1. Subjects were well matched between treatment arms, 75% were taking inhaled corticosteroids with a SABA, and 25% were taking a SABA alone. Forty-one participants produced viable induced sputum samples pre- and post-treatment (Tables 1 and 2). Medication adherence assessed by tablet counting was greater than 95% in both groups over the course of the study.

**Table 1:** Baseline demographics.

	All	PIOGLITAZONE	PLACEBO
*Number of observations*	n = 68	n = 34	n = 34
***Age (years)***	54.4 ± 13.6	51.6 ± 13.2	57.3 ± 13.7
• ***Gender****Male n* • *(%)Female n (%)*	• 37 (54.4%)31(45.6%)	• 18(52.9%)16(47.1%)	• 19(55.9%) • 15 (44.1%)
• ***Ethnicity****White n (%)Other (%)*	• 67(98.5)1(1.5)	• 33(97.01%)1 (2.9%)	34 (100%)
***Body mass index (kg/m***^***2***^***)***^†^	27.5 ± 3.6	28.1 ± 4.0	26.9 ± 3.0
***History of smoking (Pack years)*** *Median (range)*	0 (0,10)	0.1 (0,10)	0 (0,10)
***Daily inhaled steroid*** *Yes (%)No (%)*	51 (75%)17 (25%)	26 (76.5%)8 (23.5%)	25 (73.5%)9 (26.5%)
***Daily inhaled corticosteroid dose (beclomethasone diproprionate equivalent [mcg/day])*** *Median (interquartile range)*	200 (0, 400)	300 (200, 400)	200 (0, 400)
***Duration of asthma (years)*** *Median (interquartile range)*	28.5 (18, 45.5)	27 (19, 41)	30.5 (13, 46)
***Pre-bronchodilator FEV***_***1***_ ***(liters)***	2.70 ± 0.74	2.75 ± 0.73	2.65 ± 0.75
***Predicted FEV***_***1***_ ***(%)***	90 ± 15.5	92 ± 15.5	88 ± 15.6
***FVC (liters)***	3.84 ± 1	3.92 ± 1	3.76 ± 1
***Predicted FVC (%)***	105.1 ± 15.7	107.8 ± 14.3	102.3 ± 16.8
***FEV1/FVC (%)***	70.90 ± 9.6	71 ± 10.6	70.8 ± 8.6
***FEV***_***1***_ ***reversibility at screening***^***¶***^ ***(%)*** *Median (interquartile range)*	11.3 (7, 15)^†^	11.5 (7, 15)	11.3 (6.0, 17.0)^‡^
***Exhaled nitric oxide FeNO at 50 ml flow (ppb)*** *Geometric mean (95% CI)*	24.9 (21.1, 29.4)	26.6 (20.6, 34.5)	23.20 (18.7, 28.8)
***Airway hyperresponsiveness to methacholine PD***_***20***_ ***FEV***_***1***_ (mcg)*Geometric mean (95% CI)	97.7 (59.9, 159.5)	86.5 (39.3, 190.1)^∫^	109.3 (56.7, 219.3)^∫∫^
***Juniper ACQ*^*#*^*score at baseline***	0.9 ± 0.4^~^	1.0 ± 0.7	0.9 ± 0.4^∞^
***Juniper AQLQ*^*+*^*score at baseline***	6.2 ± 0.4^~^	6.1 ± 0.6	6.2 ± 0.6^∞^
***Mean morning peak expiratory flow*^*1*^**	392 ± 95	391 ± 96	392 ± 95
***Mean evening peak expiratory flow*^*2*^**	396 ± 97	399 ± 93	396 ± 97
***Peak expiratory flow variability^*** ***(%)*** *Median (interquartile range)*	19 (15, 24)	18 (13, 27)	18 (15, 23)
***Short-acting beta***_***2***_***-agonist use during run-in (actuations/day)***	0.7 ± 1	1.5 ± 1.8	0.7 ± 1
***Sputum differential eosinophil count %*** *Geometric mean (95% CI)*^*δ*^	0.8 (0.5, 1.4)	1.2 (0.6, 2.5)	0.5 (0.2, 1.1)

Arithmetic mean values reported as mean unless specified

± Standard deviation

Geometric means are reported with a 95% confidence Interval (CI)

* PD_20_-The provocative dose of methacholine (in micrograms) that results in a 20% fall in the FEV_1_, available in 46 participants at baseline and post-treatment. A PD_20_ of 100 mcg is equivalent to a PC_20_ of 1 mg/mL

¶ The percentage change in the pre-bronchodilator FEV_1_ 15 minutes after 200 mcg of inhaled salbutamol

+ Juniper Asthma Control Questionnaire- A validated questionnaire providing a numerical assessment of asthma control over the preceding 7 days. The mean score ranges from 0 (fully controlled) to 6 (poorly controlled).

# Juniper Asthma Quality of Life Questionnaire-The standardized 32-question AQLQ assesses the impact of asthma on 4 domains: symptoms, activity limitation, emotional function, and environmental stimuli. Mean score ranges from 1 (impaired quality of life) to 7 (no impairment of quality of life).

^1 and 2^ The mean peak expiratory flow calculated from the 14-day run-in period

^Peak expiratory flow variability calculated as the difference between the highest and lowest peak expiratory flow expressed as a percentage of the mean peak expiratory flow calculated from the 14-day run-in period

FEV_1-_ *Forced expiratory volume in one second*. FVC- *Forced vital capacity*. ppb- *Parts per billion*

^†^ n = 68

^‡^ n = 34

∫ n = 22

∫∫n = 24

∞ n = 32

~ n = 66

δ n = 41 (23 pioglitazone and 18 placebo)

**Table 2:** Induced sputum indices at baseline.

Mean (SD)	n = 41	PIOGLITAZONE n = 23			PLACEBO n = 18		
	*All*	n = 23	SABA = 7	ICS = 16	n = 18	SABA = 6	ICS = 12
***Sputum total cell count*** *×10*^*6*^*/g*	1.1 ± 1.7	1.0 ± 1.2	1.3 ± 1.4± 1.4	0.9 ± 1.2	1.3 ± 2.6	0.6 ± 0.4	1.7 ± 2.6
***Sputum differential neutrophil count %***	49.0 ± 26.1	47.3 ± 26.3	64.6 ± 20.7	39.7 ± 25.4	51.2 ± 26.3	46.3 ± 35.4	53.6 ± 22
***Sputum differential eosinophil count*** *% Geometric mean (95% CI)*^*¶*^	0.8 (0.5, 1.4)	1.2 (0.6, 2.5)	0.4 (0.08, 2.4)	1.8 (0.8, 4.2)	0.5 (0.2, 1.1)	0.4 (0.07, 2.4)	0.5 (0.2, 1.5)
***Sputum differential macrophage count*** *%*	43.1 ± 26.0	41.8 ± 27.1	29.2 ± 20	47.4 ± 28.5	44.6 ± 25.3	47.8 ± 32.9	43.0 ± 22.0
***Sputum differential lymphocyte count*** *%*	0.6 ± 0.6	0.7 ± 0.7	0.8 ± 1.0	0.6 ± 0.6	0.4 ± 0.4	0.4 ± 0.4	0.4 ± 0.4
***Sputum differential epithelial cell count*** *%*	3.9 ± 4.9	5.0 ± 5.5	3.9 ± 3.8	5.5 ± 6.1	2.4 ± 3.6	4.2 ± 5.7	1.5 ± 1.7
***Sputum squamous contamination*** *%*	4.2 ± 4.1	4.5 ± 4.8	4.0 ± 4.1	4.7 ± 5.2	3.9 ± 3.2	4.10 ± 2.0	3.8 ± 3.7

Values reported as arithmetic mean unless specified

± Standard deviation

¶ Geometric mean reported with a 95% confidence interval (CI)

SABA- Short-acting β_2_-agonist

ICS- Inhaled corticosteroid

### Closure of recruitment

The European Medicines Agency (EMA) reported that pioglitazone may be associated with an increased risk of developing bladder cancer in July 2011 (20). Because there were no proven benefits associated with taking pioglitazone in asthma, the TSC concluded that the risk to benefit ratio had become unacceptable for participants with mild asthma and recruitment was stopped on 14 October, 2011. At that point, 68 of the minimum requirement of 88 participants had been randomized, with 52 participants having completed the study and 10 still in the treatment phase of the study. Among those still participating in the study, 70% withdrew after receiving notification of the EMA report, while three participants elected to complete the study. All participants and investigators remained blinded to allocation until the analysis was completed.

### Efficacy

**Fig 2:**
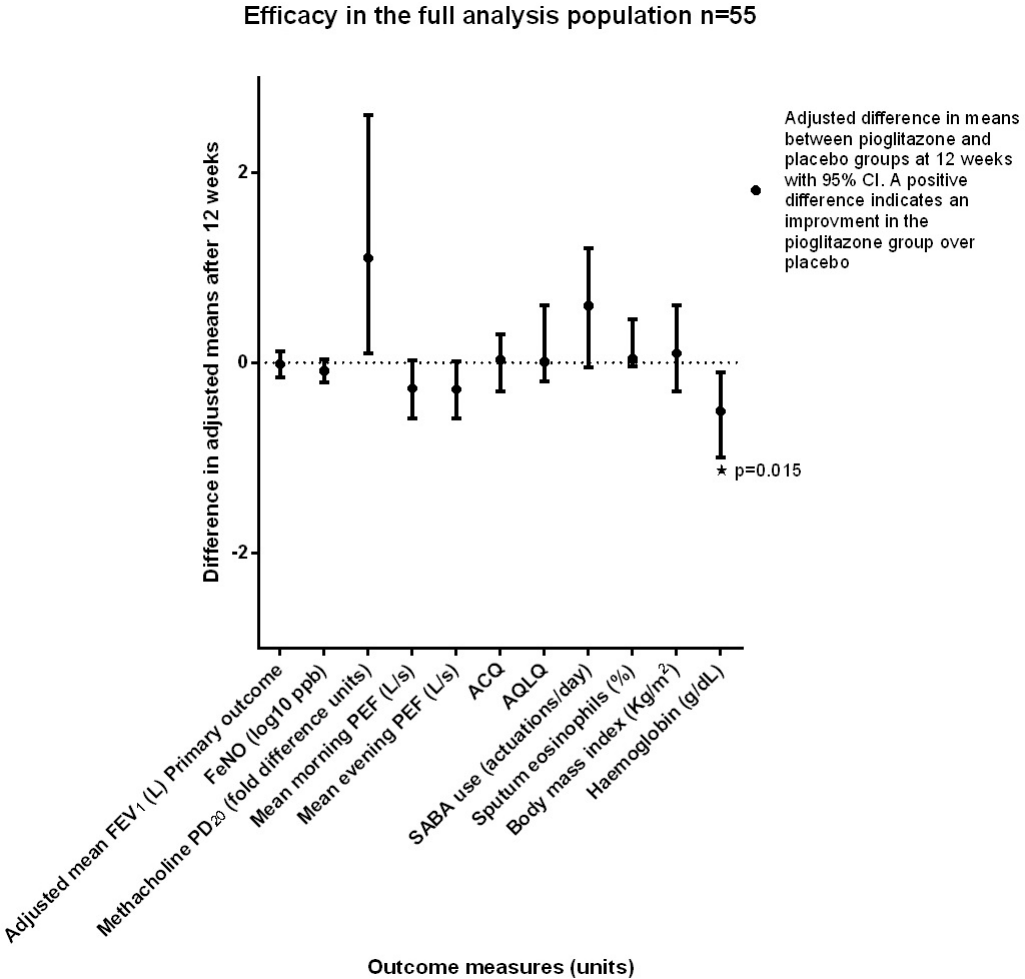
Efficacy analysis. The adjusted mean of the secondary clinical outcomes at 12 weeks was compared between groups using ANCOVA including terms for baseline value, treatment arm, inhaled corticosteroid use, age, gender, and height and expressed as the difference in the adjusted means with a 95% confidence interval (CI). FEV1- Forced expiratory volume in one second. FeNO–Fractional exhaled nitric oxide. ppb—Parts per billion. PD_20_—The provocative dose of methacholine (in micrograms) that results in a 20% fall in the FEV1, available in 46 participants at baseline and post-treatment. A PD_20_ of 100 mcg is equivalent to a PC_20_ of 1 mg/mL. Fold difference units—antilog of the (adjusted mean difference) with 95% confidence interval. A 1-fold change is equivalent to 0 doubling doses. The mean peak flow calculated from the 14 days prior to the week 12 visit. ACQ—Juniper Asthma Control Questionnaire—A validated questionnaire providing a numerical assessment of asthma control over the preceding 7 days. The mean score ranges from 0 (fully controlled) to 6 (poorly controlled). AQLQ—Juniper Asthma Quality of Life Questionnaire—The standardized 32-question AQLQ assesses the impact of asthma on 4 domains: symptoms, activity limitation, emotional function, and environmental stimuli. Mean score ranges from 1 (impaired quality of life) to 7 (no impairment of quality of life). SABA–Short acting beta_2_ agonist. Induced sputum data available for 41 participants in the full analysis.

There was no significant difference observed in the primary outcome measure, the adjusted mean FEV_1_ at 12 weeks, between the pioglitazone and placebo groups (adjusted mean difference -0.014 L, 95% CI -0.15 to 0.12, p = 0.84). In the FA there were no differences in any of the secondary outcomes between the groups at 12 weeks, although there was a trend toward a lower adjusted mean morning PEF (-16 L/min 95% CI -33 to 2, p = 0.075) and evening PEF (-17 L/min, 95% CI -35 to 1, p = 0.068) in the pioglitazone group. There was also a trend toward a higher use of as required SABA use in the pioglitazone group (0.6 actuations/day, 95% CI -0.05 to 1.2, p = 0.07) (Fig 2 and Table 3).

**Table 3:** Full analysis.

	PIOGLITAZONE n = 27			PLACEBO n = 28				
	Baseline *(SD)*	Week 12	Adjusted mean at 12 weeks (95% CI)	Baseline	Week 12	Adjusted mean at 12 weeks	Adjusted difference in means at 12 weeks between group(95% CI)	p-value (95% CI)
***FEV***_***1***_ ***(liters)***	2.71 ± 0.82.71	2.75 ± 0.82.75	2,67 (2.58, 2.76)	2.59 ± 0.82.59	2.61 ± 0.82.61	2.69 (2.60, 2.78)	**-0.014 (-0.15, 0.12)-0.014**	0.84
***Exhaled nitric oxide FeNO (ppb)***^***¶***^ *geometric mean (95% CI)*	31.6 (24, 41.7)	24.0 (18.6, 30.2)	21.9 (18.2, 26.3)	23.4 (18.2, 29.5)	24.6 (19.5, 31.6)	26.6 (22.1, 31.8)	**0.8 (0.6, 1.1)**	0.18
***Airway hyperresponsiveness to methacholine PD***_***20***_ ***FEV***_1_* ***mcg*. *Adjusted difference fold difference units^*** ***(95% CI)***	86.5 (38.9, 190.6)	114.8 (55, 239.9)	129.2 (74.1, 223.9)	109.3 (56.7, 210.9)	128 (57.5, 284.8)	114.4 (67.6, 195)	**1.1^ (0.1, 2.6)**	0.77
***Mean morning PEF***^***1***^ *(L/min)*	385 ± 99	388 ± 106	388 (376, 400)	392 ± 99	404 ± 109	404 (392, 416)	**-16 (-35, 2)**	0.075
***Mean evening PEF***^***2***^ *(L/min)*	393 ± 96	394 ± 107	390 (379, 402)	395 ± 101	404 ± 108	407 (395, 419)	**-17 (-35, 1)**	0.068
***Juniper ACQ***^***+***^	1.1 ± 0.7	0.7 ± 0.5	0.7 (0.5, 0.9)	0.9 ± 0.3	0.7 ±0.5	0.7 (0.5, 0.9)	**0.03 (-0.3, 0.3)**	0.86
***Juniper AQLQ***^***#***^	6.0 ± 0.7	6.3 ± 0.6	6.3 (6.1, 6.5)	6.1 ± 0.6	6.3 ±0.6	6.3 (6.1, 6.4)	**0.01 (-0.2, 0.6)**	0.94
***Short-acting beta***_***2***_***-agonist use in the 14 days preceding week 12*** *(actuations/day)*	1.7 ± 1.8	1.1 ± 1.5	1.1 (0.7, 1.4)	0.8 ± 1	0.4 ±0.5	0.5 (0.1, 0.9)	**0.6 (-0.05,1.2)**	0.07
***Body mass index at week 12*** *(kg/m*^*2*^*)*	27.5 ± 3.1	27.8 ± 3.1	27.4 (27.1, 27.6)	26.8 ± 3.2	26.9 ±3.3	27.3 (27, 27.6)	**0.06 (-0.4, 0.5)**	0.76

Values are reported as arithmetic mean unless specified

± Standard deviation

¶ Geometric means are reported with a 95% confidence interval (CI)

The adjusted mean of the secondary clinical outcomes at 12 weeks was compared between groups using ANCOVA including terms for baseline value, treatment arm, inhaled corticosteroid use, age, gender, and height and expressed as the difference in the adjusted means with a 95% confidence interval (CI) and p-value

*PD_20_-The provocative dose of methacholine (in micrograms) that results in a 20% fall in the FEV_1_, available in 46 participants at baseline and post-treatment.

A PD_20_ of 100 mcg is equivalent to a PC_20_ of 1 mg/ml

^Fold difference units- antilog of the (adjusted mean difference) with 95% CI. A 1 fold change is equivalent to 0 doubling doses

+ Juniper Asthma Control Questionnaire- A validated questionnaire providing a numerical assessment of asthma control over the preceding 7 days. The mean score ranges from 0 (fully controlled) to 6 (poorly controlled).

# Juniper Asthma Quality of Life Questionnaire-The standardized 32-question AQLQ assesses the impact of asthma on 4 domains: symptoms, activity limitation, emotional function, and environmental stimuli. Mean score ranges from 1 (impaired quality of life) to 7 (no impairment of quality of life).

^1 and 2^ The mean peak flow calculated from the 14 days prior to the week 12 visit

FEV_1-_ Forced expiratory volume in one second

FVC- Forced vital capacity

ppb- Parts per billion

FEV_1-_ Forced expiratory volume in one second

ANCOVA- Analysis of covariance

The per protocol analysis replicated the full analysis, except that the mean evening PEF was significantly lower in the pioglitazone group after 12 weeks (-21 L/min, 95% CI -39 to -4, p = 0.02) (Table 4).

**Table 4:** Per-protocol analysis.

	PIOGLITAZONE n = 27			PLACEBO n = 28				
	Baseline *(SD)*	Week 12	Adjusted mean at 12 weeks (95% CI)	Baseline	Week 12	Adjusted mean at 12 weeks	Adjusted difference in means at 12 weeks between group (95% CI)	p-value (95% CI)
***FEV***_***1***_ ***(liters)***	2.76 ± 0.8	2.80 ± 0.8	2.68 (2.59, 2.78)	2.58 ± 0.8	2.60 ± 0.8	2.71 (2.61, 2.81)	-0.03 (-0.17, 0.12)	0.71
***Exhaled nitric oxide FeNO (ppb)***^***¶***^ *geometric mean (95% CI)*	32.7 (25, 42.8)	23.8 (18.5, 30.5)	22.2 (18.3, 27)	25.1 (19.8, 31.8)	25.9 (20.4, 33.1)	27.9 (22.8, 33.6)	0.8 (0.6, 1.1)	0.14
***Airway hyperresponsiveness to methacholine PD***_***20***_ ***FEV***_1_* ***Adjusted difference in means fold difference units (95% CI)***	87.8 (38, 201)	114.2 (52.5, 248.3)	123.2 (71.3, 212.9)	106.3 (53, 213.3)	133.6 (56.6, 315.5)	124.2 (72.9, 211.7)	1.0^ (0.4, 2.3)	0.98
***Mean morning PEF***^***1***^ *(L/min)*	389 ± 99	393 ± 105	390 (378, 402)	393 ± 99	406 ± 110	409 (396, 421)	-19 (-38, 0.3)	0.053
***Mean evening PEF***^***2***^ *(L/min)*	397 ± 95	399 ± 106	391 (379, 402)	393 ± 98	404 ± 109	412 (400, 424)	-21 (-39, 4)	**0.02**
***Juniper ACQ***^***+***^	1.1 ± 0.7	0.7 ± 0.6	0.7 (0.6, 0.9)	0.9 ± 0.3	0.6 ± 0.4	0.6 (0.4, 0.7)	0.2 (-0.1, 0.4)	0.23
***Juniper AQLQ***^***#***^	6.0 ± 0.7	6.3 ± 0.6	6.3 (6.2, 6.5)	6.3 ± 0.4	6.4 ± 0.5	6.4 (6.2, 6.5)	-0.1 (-0.3, 0.2)	0.69
***Short-acting beta***_***2***_***-agonist use in the 14 days preceding week 12*** *(actuations/day)*	1.6 ± 1.7	1.0 ± 1.4	1.0 (0.6, 1.4)	0.8 ± 1.0	0.4 ± 0.5	0.4 (0.2,0.80)	0.6 (-0.01, 1.2)	0.055
***Body mass index at week 12*** *(kg/m*^*2*^*)*	27.3 ± 2.9	27.5 ± 2.9	27.3 (27, 27.5)	26.8 ± 3.24	26.8 ± 3.3	27.1 (26.8, 27.4)	0.1 (-0.3, 0.6)	0.51

Values are reported as arithmetic mean unless specified

± Standard deviation

¶ Geometric means are reported with a 95% confidence interval (CI)

The adjusted mean of the secondary clinical outcomes at 12 weeks was compared between groups using ANCOVA including terms for baseline value, treatment arm, inhaled corticosteroid use, age, gender, and height and expressed as the difference in the adjusted means with a 95% confidence interval (CI) and p-value.

*PD_20_-The provocative dose of methacholine (in micrograms) that results in a 20% fall in the FEV_1_, available in 43 participants at baseline and post-treatment.

A PD_20_ of 100 mcg is equivalent to a PC_20_ of 1 mg/mL.

^Fold difference units- antilog of the (adjusted mean difference) with 95% CI. A 1-fold change is equivalent to 0 doubling doses.

+ Juniper Asthma Control Questionnaire- A validated questionnaire providing a numerical assessment of asthma control over the preceding 7 days. The mean score ranges from 0 (fully controlled) to 6 (poorly controlled).

# Juniper Asthma Quality of Life Questionnaire-The standardized 32-question AQLQ assesses the impact of asthma on 4 domains: symptoms, activity limitation, emotional function, and environmental stimuli. Mean score ranges from 1 (impaired quality of life) to 7 (no impairment of quality of life).

^1 and 2^ The mean peak flow calculated from the 14 days prior to the week 12 visit

FEV_1-_ Forced expiratory volume in one second

FVC- Forced vital capacity

ppb- Parts per billion

FEV_1-_ Forced expiratory volume in one second

ANCOVA- Analysis of covariance

**Fig 3:**
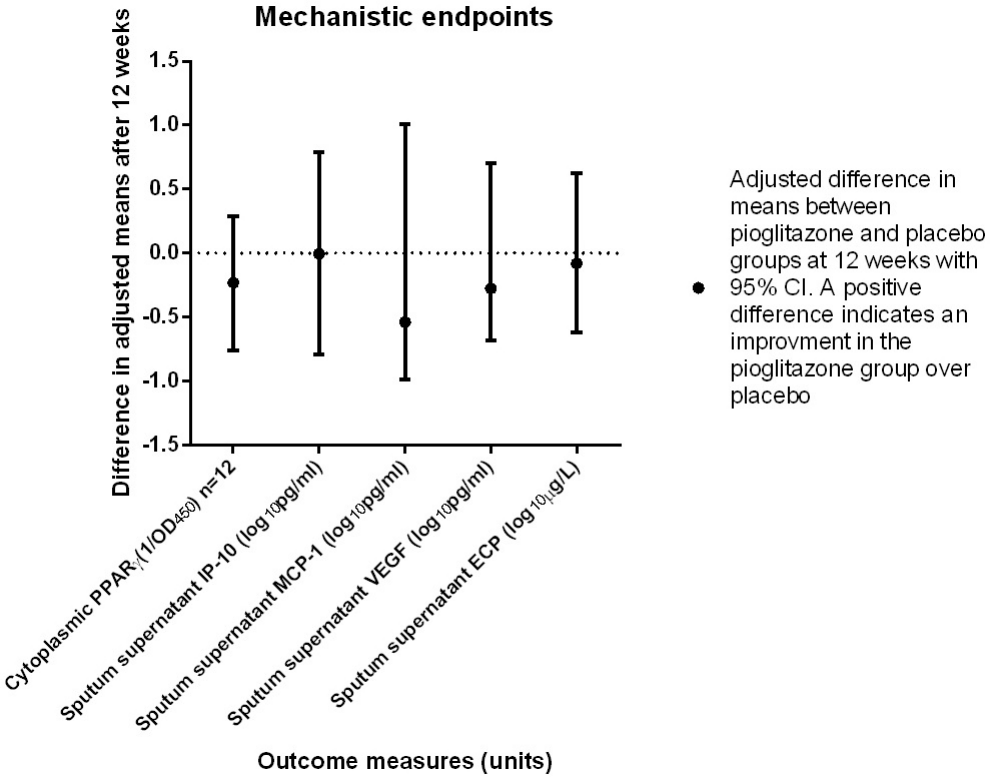
Mechanistic endpoints. In subsets of participants, the following assays were performed: Cytoplasmic PPAR-γ transcription factor assay. Pioglitazone n = 6, placebo n = 6. Mean of the reciprocal OD450. IP-10, MCP-1, VEGF and levels in sputum supernatant, assay (Bio-Plex) pg/mL normalized to 1 x 10^6^ sputum cells. Pioglitazone n = 11, placebo n = 8. ECP pioglitazone n = 10 (total ECP n = 18) Phadia ImmunoCAP normalized to 1 x 10^6^ sputum cells. PPAR-γ- Peroxisome proliferator-activated receptor gamma. OD Optical density (spectrophotometry). IP-10- Interferon gamma-inducible protein-10. VEGF- Vascular endothelial growth factor. MCP-1- Monocyte chemotactic protein-1. ECP- Eosinophil cationic protein.

In subsets of participants that provided induced sputum samples pre- and post-treatment, there were no significant differences in the induced sputum differential cell counts or the induced sputum supernatant ECP, MCP-1, IP-10, or VEGF, or the nuclear and cytoplasmic levels of PPAR-γ between groups (Figs 2 and 3).

There was an increase in the reporting of expected side effects of pioglitazone in the active group, particularly ankle swelling, weight gain, insomnia, and lethargy (Table 5). There were no asthma exacerbations requiring oral corticosteroids or hospital admission in the study; however, five participants in the pioglitazone group and two in the placebo group reported mild/moderate asthma exacerbations that required an increase in SABAs.

**Table 5:** Reported adverse events occurring in more than 1 participant by system.

	PIOGLITAZONE	PLACEBO
***Respiratory system***		
*Upper respiratory tract infection*	8	8
*Exacerbation of asthma (mild/moderate)*	5	2
***Blood and lymphatic disorders***		
*Thrombocytopenia*	2	0
*Leukopenia or Neutropenia*	1	1
***Nervous system disorders***		
*Insomnia*	5	1
*Alteration of mood*	3	0
*Headache*	1	4
***Eye disorders***		
*Hordeolum*	2	0
***Ear and labyrinth disorders***		
*Epistaxis*	2	1
*Vertigo*	0	2
***Renal tract***		
*Urinary tract infection*	0	2
***Musculoskeletal system***		
*Arthralgia*	1	1
***General disorders***		
*Ankle edema (spontaneously reported)*	8	2
*Weight gain (spontaneously reported)*	4	0
*Fatigue*	3	0
***Investigations***		
*Weight increased (> 5% of baseline)*	3	1

The pioglitazone-treated group had a significantly lower adjusted mean hemoglobin (-0.51 g/dL, 95% CI -1 to -0.1, p = 0.015), red cell count (-0.2 x 10^12^/L, 95% CI -0.03 to -0.04, p = 0.012) and adjusted mean cell hemoglobin (-0.6 g/dL, 95% CI -1.1 to -0.08, p = 0.024) compared with the placebo-treated group at week 12 (Fig 2 and Table 6).

**Table 6:** Safety set blood test monitoring.

	PIOGLITAZONE			PLACEBO				
	*Baseline n = 34 (SD)*	*Week 12 (n = 28)*	*Week 12 adjusted mean n = 27 (95% CI)*	*Baseline n = 34 (SD)*	*Week 12 (n = 29)*	*Week 12 adjusted mean (95% CI)*	Difference in adjusted means between groups at 12 weeks	p-value (95% CI)
***Hemoglobin*** *(g/dL)*	13.9 ± 1.2	13.3 ± 1.3	13.5 (13.2, 13.8)	14.3 ± 1.1	14.3 ± 1.1	14.1 (13.8, 14.3)	-0.51 (-1, -0.1)	0.015*
***Hematocrit***	0.4 ± 0.03	0.41 ± 0.03	0.4 (0.4,0.4)	0.4 ± 0.02	0.4 ± 0.03	0.4 (0.4, 0.4)	-0.01 (-0.03, 0.0003)	0.056
***Mean cell hemoglobin concentration*** *(g/dL)*	33.1 ± 1.2	32.6 ± 1.1	32.8 (32.5, 33.1)	33.4 ± 1.1	33.6 ± 0.9	33.4 (33, 33.7)	-0.6 (-1.1, -0.08)	0.024*
***White cell count*** *x 10*^*9*^*/L*	6.8 ± 1.7	6.4 ± 1.6	6.5 (6, 6.9)	6.9 ± 1.6	6.8 ± 1.8	6.7 (6.3, 7.2)	-0.3 (-1, 0.5)	0.45
***Platelet count*** *x 10*^*9*^*/L*	246 ± 50	237 ± 53	233 (219, 247)	239 ± 41	244 ± 50	248 (235, 262)	-15 (-36, 5)	0.14
***Mean cell volume (MCV)*** *fL*	89 ± 5	90 ± 6	90 (89, 91)	90 ± 4	90 ± 4	90 (89, 90)	0.7 (-0.5, 1.9)	0.27
***Blood neutrophils*** *x 10*^*9*^*/L*	3.9 ± 1.5	3.7 ± 1.3	3.8 (3.3, 4.2)	3.9 ± 1.1	3.9 ± 1.6	3.8 (3.4, 4.3)	-0.09 (-0.7, 0.6)	0.79
***Log***_***10***_ ***blood eosinophils*** *x 10*^*9*^*/L*	-0.7 ± 0.2	-0.8 ± 0.2	-0.8 (-0.9, -0.7)	-0.7 ± 0.3	-0.7 ± 0.2	-0.7 (-0.8, -0.6)	-0.09 (-0.2, 0.02)	0.11
***Log***_***10***_ ***ALT*** *U/L*	1.4 ± 0.2	1.3 ± 0.3	1.3 (1.3, 1.4)	1.3 ±0.2	1.3 ±1.3	1.3 (1.3, 1.4)	-0.02 (-0.09, 0.04)	0.49
***Log***_***10***_ ***GGT***	1.41 ±0.25	1.31 ±0.27	1.30 (1.26, 1.33)	1.36 ± 0.16	1.36 ± 0.16	1.37 (1.33, 1.40)	-0.07 (-0.12, -0.024)	0.004*
***Total bilirubin*** *μmol/L*	9.1 ± 3.6	9.7 ± 3.9	10.1 (9.1, 11.1)	10.1 ± 3.7	10.1 ± 3.7	9.84 (8.9, 10.8)	0.2 (-1.2, 1.7)	0.76
***Total alkaline phosphatase*** *U/L*	69.1 ± 21.1	63.3 ± 18.9	63.8 (61.2, 66.4)	72.4 ± 15.1	70.6 ± 11	70 (67.5, 72.6)	-6.2 (-10.1, -2.4)	0.002*
***Random glucose*** *mmol/L*	5.2 ± 0.8	5.0 ± 0.9	5.1 (4.7, 5.4)	5.5 ± 1.1	5.4 ± 0.8	5.4 (5.1, 5.7)	-0.3 (-0.8, 0.2)	0.21

*p value < 0.05

Values reported as arithmetic mean unless specified

± Standard deviation

The adjusted mean of the secondary outcomes at 12 weeks was compared between groups using ANCOVA including terms for baseline value, treatment arm, inhaled corticosteroid use, age, gender, and height and expressed as the difference in the adjusted means with a 95% CI and p-value.

ALT- alanine aminotransferase. GGT- gamma glutamyl transferase. ANCOVA- analysis of covariance

## Discussion

In this randomized controlled trial, the administration of pioglitazone for 12 weeks in mild asthma did not result in any improvements in a range of markers of asthma control or measures of airway inflammation. In fact, we observed a lower post-treatment mean evening PEF and a trend toward higher SABA reliever use in the pioglitazone group, which was not accompanied by significant deterioration in asthma control or scores on quality of life questionnaires. Although these findings most likely reflect the multiplicity of analyses, they could have been caused indirectly by side effects of pioglitazone such as insomnia and fatigue. If the reduction in PEF is a true effect, and assuming a minimum important difference (MID) for PEF of 15 to 20 L/minute, this is of a magnitude that could have been perceived by the participants [28].

Despite evidence that PPAR-γ activation has anti-inflammatory effects in experimental models of asthma, there are a number of potential reasons why pioglitazone may not have exerted an effect in the present study. Firstly, the early closure of recruitment limited the number of participants completing the study, leaving the study underpowered to detect a difference of 150 ml between groups. However, considering the narrow 95% CIs for the difference in FEV_1_, the lack of change in airway hyper-responsiveness, lower PEF, and trend toward higher SABA use in the pioglitazone group, it is unlikely that we missed a clinically important effect on airway dysfunction in mild asthma.

It is possible that the observed lack of efficacy was because pioglitazone was not biologically active. Although we did not measure plasma levels of pioglitazone, more than 95% of the study capsules were taken by participants and we observed a decrease in hemoglobin, a recognized systemic side effect of pioglitazone [29–31]. However, despite evidence of systemic biological activity, we were unable to confirm that pioglitazone successfully activated PPAR-γ in the lungs to induce nuclear translocation of PPAR-γ within the induced sputum cells. As pioglitazone is highly bound to plasma proteins (>99%) and has a low volume of distribution across tissues, it may not have entered the airways in sufficient concentration to exert an effect.

An alternative explanation for the lack of efficacy is that the study participants had adequate asthma control with low levels of eosinophilic airway inflammation and preserved lung function at baseline, leaving little potential for improvement. Recruiting patients with evidence of eosinophilic inflammation would have been better but would have rendered recruitment into the study even more difficult. Therefore, we have not excluded a possible anti-inflammatory effect in patients with more severe asthma and more marked airway inflammation, and a study with longer duration in this cohort would be required to assess the effect of pioglitazone on the risk of asthma exacerbations.

To our knowledge, this is the largest and longest study of a PPAR-γ agonist in asthma performed to date [16– 19]. A previously reported RCT compared rosiglitazone to inhaled corticosteroids in 46 current smokers with asthma (a group associated with poor response to inhaled corticosteroids), and reported a trend toward a higher pre-bronchodilator FEV_1_ in the group treated with rosiglitazone at 28 days (183 mL, 95% CI -1.0 to 367, p = 0.051) (18). There was also an improvement in the forced expiratory flow between 25 and 75% of the forced vital capacity after 28 days of treatment with rosiglitazone (FEF_25-75_ 0.24 L/second, 95% CI 0.03 to 0.46, p = 0.03), but no statistically significant improvements in ACQ scores or sputum cell counts [18]. A second RCT compared 28 days of treatment with rosiglitazone with placebo in 32 adults with mild asthma and reported a 15% attenuation of the late asthmatic reaction, with no change in the early asthmatic reaction compared to placebo. The authors also reported no effect on airway hyper-responsiveness after allergen challenge or on a panel of inflammatory markers [17]. Dixon and colleagues reported a 12 week RCT comparing pioglitazone (n = 23) to placebo (n = 19) in obese subjects with poorly controlled moderate to severe asthma [19]. Using 30mg pioglitazone for 2 weeks increased to 45mg for 10 weeks there were no significant differences in the airway hyper-responsiveness to methacholine, FeNO or lung function between treatment and placebo. Recruitment was halted early (as in our study) due to the impact of emerging safety concerns surrounding the potential risk of bladder cancer affecting recruitment, lack of efficacy signal and significant weight gain in pioglitazone treated patients [19]. The thiazolidinediones are associated with significant adverse effects when administered systemically and these studies support our conclusion that systemic treatment with pioglitazone is not associated with beneficial effects on asthma control.

## Conclusion

We found no evidence to support the hypothesis that 12 weeks of treatment with the PPAR-γ agonist pioglitazone improves asthma control or airway inflammation in individuals with mild asthma. Opportunities to further investigate the potential anti-inflammatory effects of pioglitazone in more severe asthma are currently limited by safety considerations.

## Supporting Information

S1 Data Full trial dataset. (XLSX)

S1 Text Protocol. (DOC)

S2 Text CONSORT checklist. (DOC)
