# Supplementary material for: Evaluation of the PPAR-γ Agonist Pioglitazone in Mild Asthma: A Double-Blind Randomized Controlled Trial
Source: PLoS One. 2016 Aug 25;11(8):e0160257. doi: 10.1371/journal.pone.0160257 (PMC4999189; doi:10.1371/journal.pone.0160257)
Supplement: S1 Text — (DOC) [file pone.0160257.s002.doc]

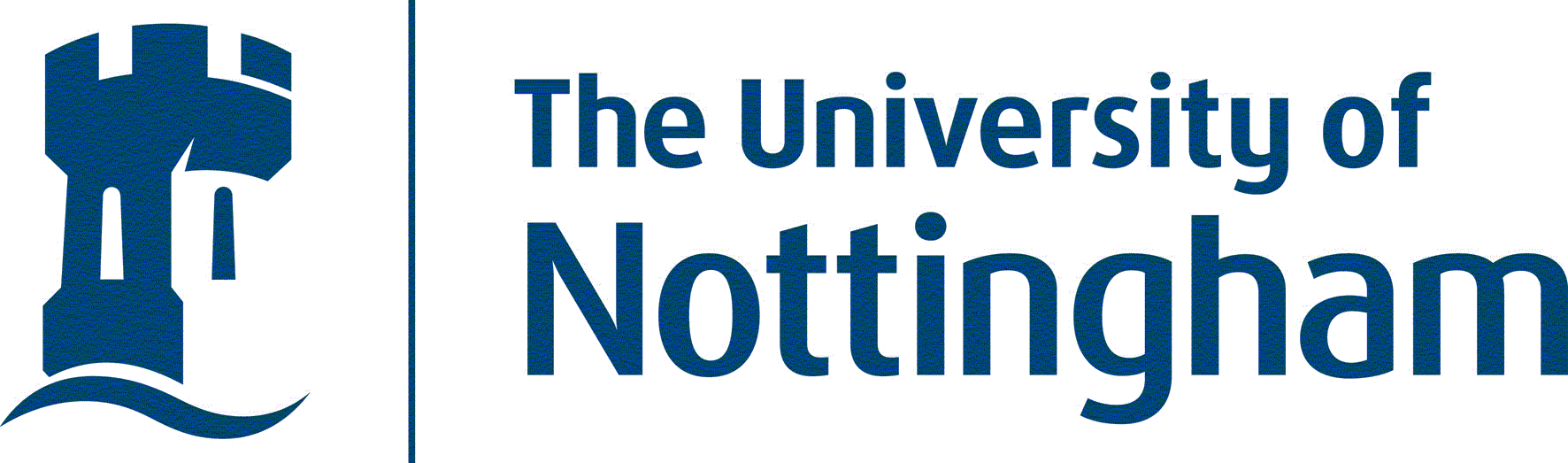


**PPAR-γ: a novel therapeutic target for asthma?**

**Final Version 5.0**

**10^th^ February 2010**

**Short title:** PPAR-γ: a novel therapeutic target for asthma?

**Acronym:** N/A

**EudraCT number:** 2008-004112-13

**Trial Registration:** [www.clinicaltrials.gov](http://www.clinicaltrials.gov/) reference

**ISRCTN:** *as appropriate*

**CTA reference:** *as appropriate*

**MREC reference:** 08/H0408/120

**Trial Sponsor:** University of Nottingham

**Sponsor protocol No:** 33100

**Funding Source:** Efficacy and Mechanism Evaluation Programme

Nottingham Biomedical Research Unit

TRIAL / STUDY PERSONNEL AND CONTACT DETAILS

**Sponsor:** University of Nottingham

Contact name Mr Paul Cartledge

Head of Research Grants and Contracts

Research Innovation Services

King’s Meadow Campus

Lenton Lane

Nottingham

NG7 2NR

**Chief investigator:** Dr Tim Harrison

(medical expert) Respiratory Division

Clinical Sciences Building

City Hospital

Nottingham

NG5 1PB

Phone: 0115 9691169 x 56317 (sec) x54177

Fax: 0115 8231946

Email: tim.harrison@nottingham.ac.uk

**Co-investigators:** Dr Kevin Mortimer

Respiratory Division

Clinical Sciences Building

City Hospital

Nottingham

NG5 1PB

Phone: 0115 8231694

Email: Kevin.mortimer@nottingham.ac.uk

Dr John Anderson

Respiratory Medicine

City Hospital

Nottingham

NG5 1PB

E-mail: john.anderson@nottingham.ac.uk

Dr Linhua Pang

Respiratory Division

Clinical Sciences Building

City Hospital

Nottingham

NG5 1PB

Email: linhua.pang@nottingham.ac.uk

Prof Alan Knox

Respiratory Division

Clinical Sciences Building

City Hospital

Nottingham

NG5 1PB

Email: alan.knox@nottingham.ac.uk

**Trial / Study Statistician:** Dr Paul Silcocks

Clinical Trials Unit

Office B39

Medical School

Queen’s Medical Centre

Nottingham NG7 2UH

Email: paul.silcocks@nottingham.ac.uk

**Trial Pharmacist:** Dr Sarah Pacey

Main Pharmacy

City Hospital

Nottingham

NG5 1PB

Phone:

Fax:

Email: S.Pacey@nuh.nhs.uk

**Trial / Study Coordinating Centre:** Nottingham University Hospitals NHS Trust

Nottingham University

**Project / Trial Manager:** N/A

# SYNOPSIS

| Title | PPAR-γ: a novel therapeutic target for asthma? |
| --- | --- |
| Acronym | N/A |
| Short title | PPAR-γ: a novel therapeutic target for asthma? |
| Chief Investigator | Tim Harrison |
| Objectives | To test the hypothesis that stimulation of PPAR-γ receptors has a therapeutic role in the treatment of asthma |
| Trial Configuration | Randomized, double blind, placebo controlled two parallel group clinical trial |
| Setting | Primary care & Secondary care |
| Sample size estimate | Assuming the SD of differences in Forced Expiratory Volume in One Second (FEV_1_ ) over 12 weeks is 250 a study with 88 participants will allow us to detect a difference between treatment arms in the mean 12 week FEV_1_ of 150 ml with 80 % power at a 5% two-sided significance level. In practice we aim to recruit 100 subjects to allow for drop-outs. |
| Number of participants | 100 |
| Eligibility criteria | The inclusion criteria will be age 18-75 of either sex with a clinical diagnosis of asthma, FEV_1_ ≥60% predicted and an increase in FEV_1_ of greater than 12% following inhaled salbutamol 400μg or Peak Expiratory Flow (PEF) variability >12% during run-in. Allowed medication: 0-800μg inhaled beclomethasone diproprionate or equivalent and as required short acting beta agonist. The exlusion criteria will be inibility to produce a sputum sample on induction current smoking, >10 pack years smoking history, treatment with leukotriene antagonists, long acting beta-agonists or theophylline, liver or cardiovascular disease, oral steroid treatment or exacerbation within 6 weeks, females who are pregnant, lactating or not using adequate contraception, any contra-indication to pioglitazone (hypersensitivity to pioglitazone, cardiac failure, history of cardiac failure, hepatic impairment, diabetic ketoacidosis), oral or insulin treatment for diabetes, treatment with gemfibrozil or rifampicin or lactose intollerance. |
| Description of interventions | Pioglitazone 30mg daily by mouth for 4 weeks then 45mg daily for 8 weeks and placebo 30mg daily by mouth for 4 weeks then 45mg daily for 8 weeks. |
| Duration of study | Planned start date 1^st^ January 2010  Anticipated duration 24 months  Duration of involvement per participant: 18 weeks |
| Randomisation and blinding | Generation of randomisation sequence by a computer generated random code using random permuted blocks of randomly varying size. Sequence to be held in pharmacy in trial folder, concealed from investigators and participants. Both investigators and participants will be blinded. |
| Outcome measures | Primary endpoint: FEV_1_ after 12 weeks treatment  Secondary endpoints: Change over 12 weeks in daily asthma symptoms, mean morning and evening PEF, Juniper asthma control questionnaire and asthma quality of life questionnaire scores, exhaled nitric oxide level, bronchial hyper-responsiveness, induced sputum cell counts and analysis detailed above, adverse effects |
| Statistical methods | The analysis of the Primary response variable (FEV_1_ at 12 weeks) will be performed using a General Linear Model incorporating terms for baseline value, treatment arm, stratum, and baseline characteristics which are thought *a priori* to strongly predict outcome.  Secondary response variables will be treated similarly, after transformation to approximate Normality as required (as, for example with PD20, Percentage eosinophil count and exhaled NO concentrations). Mean morning and evening PEFR and the percentage of days and nights free of symptoms and relief inhaler use will be calculated for the 14 days prior to each visit and analysed as above. All analyses will be performed using Stata v10. |

# ABBREVIATIONS

| ADR | Adverse Drug Reaction |
| --- | --- |
| AE | Adverse Event |
|  |  |
| CI | Chief Investigator overall |
|  |  |
| CRF | Case Report Form |
|  |  |
| DAP | Data Analysis Plan |
| DMC | Data Monitoring Committee |
|  |  |
| EMEA | European Agency for the Evaluation of Medicinal Products |
| EOT | End of Trial |
|  |  |
| GCP | Good Clinical Practice |
|  |  |
| ICF | Informed Consent Form |
|  |  |
| MHRA | Medicines and Healthcare products Regulatory Agency |
|  |  |
| NHS | National Health Service |
|  |  |
| P/GIS | Parent / Guardian Information Sheet |
| PI | Principal Investigator at a local centre |
| PIS | Participant Information Sheet |
|  |  |
| REC | Research Ethics Committee |
| R&D | Research and Development department |
|  |  |
| SAE | Serious Adverse Event |
| SAR | Serious Adverse Reaction |
| SPC | Summary of Product Characteristics |
| SUSAR | Suspected Unexpected Serious Adverse Reaction |
|  |  |
| TMG | Trial Management Group |
| TSC | Trial Steering Committee |

**TABLE OF CONTENTS**

[TRIAL / STUDY PERSONNEL AND CONTACT DETAILS 2](#__RefHeading___Toc201129564)

[SYNOPSIS 4](#__RefHeading___Toc201129565)

[ABBREVIATIONS 6](#__RefHeading___Toc201129566)

[TRIAL / STUDY BACKGROUND INFORMATION AND RATIONALE 9](#__RefHeading___Toc201129567)

[DETAILS OF INVESTIGATIONAL MEDICINAL PRODUCT(S) 12](#__RefHeading___Toc201129568)

[Description 12](#__RefHeading___Toc201129569)

[Packaging and labelling 12](#__RefHeading___Toc201129570)

[Storage, dispensing and return 13](#__RefHeading___Toc201129571)

[Placebo 13](#__RefHeading___Toc201129572)

[Known Side Effects 13](#__RefHeading___Toc201129573)

[TRIAL / STUDY OBJECTIVES AND PURPOSE 14](#__RefHeading___Toc201129574)

[PURPOSE 14](#__RefHeading___Toc201129575)

[PRIMARY OBJECTIVE 14](#__RefHeading___Toc201129576)

[SECONDARY OBJECTIVES 14](#__RefHeading___Toc201129577)

[TRIAL / STUDY DESIGN 15](#__RefHeading___Toc201129578)

[TRIAL / STUDY CONFIGURATION 15](#__RefHeading___Toc201129579)

[Primary endpoint 15](#__RefHeading___Toc201129580)

[Secondary endpoint 15](#__RefHeading___Toc201129581)

[Safety endpoints 15](#__RefHeading___Toc201129582)

[Stopping rules and discontinuation 15](#__RefHeading___Toc201129583)

[RANDOMIZATION AND BLINDING 16](#__RefHeading___Toc201129584)

[Maintenance of randomisation codes and procedures for breaking code 16](#__RefHeading___Toc201129585)

[TRIAL MANAGEMENT 17](#__RefHeading___Toc201129586)

[DURATION OF THE TRIAL / STUDY AND PARTICIPANT INVOLVEMENT 17](#__RefHeading___Toc201129587)

[End of the Trial 18](#__RefHeading___Toc201129588)

[SELECTION AND WITHDRAWAL OF PARTICIPANTS 18](#__RefHeading___Toc201129589)

[Recruitment 18](#__RefHeading___Toc201129590)

[Inclusion criteria 19](#__RefHeading___Toc201129591)

[Exclusion criteria 19](#__RefHeading___Toc201129592)

[Expected duration of participant participation 19](#__RefHeading___Toc201129593)

[Removal of participants from therapy or assessments 19](#__RefHeading___Toc201129594)

[Informed consent 20](#__RefHeading___Toc201129595)

[TRIAL / STUDY TREATMENT AND REGIMEN 20](#__RefHeading___Toc201129596)

[Compliance 22](#__RefHeading___Toc201129597)

[Accountability for drugs & placebos 22](#__RefHeading___Toc201129598)

[Management of study drug overdose 22](#__RefHeading___Toc201129599)

[Criteria for terminating trial 22](#__RefHeading___Toc201129600)

[STATISTICS 24](#__RefHeading___Toc201129601)

[Methods 24](#__RefHeading___Toc201129602)

[Sample size and justification 24](#__RefHeading___Toc201129603)

[Assessment of efficacy 24](#__RefHeading___Toc201129604)

[Assessment of safety 24](#__RefHeading___Toc201129605)

[Procedures for missing, unused and spurious data 24](#__RefHeading___Toc201129606)

[Definition of populations analysed 25](#__RefHeading___Toc201129607)

[ADVERSE EVENTS 26](#__RefHeading___Toc201129608)

[Definitions 26](#__RefHeading___Toc201129609)

[Causality 27](#__RefHeading___Toc201129610)

[Reporting of adverse events 28](#__RefHeading___Toc201129611)

[SUSARs 28](#__RefHeading___Toc201129612)

[Participant removal from the study due to adverse events 29](#__RefHeading___Toc201129613)

[ETHICAL AND REGULATORY ASPECTS 29](#__RefHeading___Toc201129614)

[ETHICS COMMITTEE AND REGULATORY APPROVALS 29](#__RefHeading___Toc201129615)

[INFORMED CONSENT AND PARTICIPANT INFORMATION 29](#__RefHeading___Toc201129616)

[RECORDS 30](#__RefHeading___Toc201129617)

[Drug accountability 30](#__RefHeading___Toc201129618)

[Case Report Forms 30](#__RefHeading___Toc201129619)

[Source documents 31](#__RefHeading___Toc201129620)

[Direct access to source data / documents 31](#__RefHeading___Toc201129621)

[DATA PROTECTION 31](#__RefHeading___Toc201129622)

[QUALITY ASSURANCE & AUDIT 32](#__RefHeading___Toc201129623)

[INSURANCE AND INDEMNITY 32](#__RefHeading___Toc201129624)

[TRIAL CONDUCT 32](#__RefHeading___Toc201129625)

[TRIAL DATA 32](#__RefHeading___Toc201129626)

[RECORD RETENTION AND ARCHIVING 33](#__RefHeading___Toc201129627)

[DISCONTINUATION OF THE TRIAL BY THE SPONSOR 33](#__RefHeading___Toc201129628)

[STATEMENT OF CONFIDENTIALITY 33](#__RefHeading___Toc201129629)

[PUBLICATION AND DISSEMINATION POLICY 34](#__RefHeading___Toc201129630)

[USER AND PUBLIC INVOLVEMENT 34](#__RefHeading___Toc201129631)

[STUDY FINANCES 34](#__RefHeading___Toc201129632)

[Funding source 34](#__RefHeading___Toc201129633)

[Participant stipends and payments 34](#__RefHeading___Toc201129634)

[REFERENCES 35](#__RefHeading___Toc201129635)

[SIGNATURE PAGES 39](#__RefHeading___Toc201129636)

# TRIAL / STUDY BACKGROUND INFORMATION AND RATIONALE

Chronic asthma causes significant morbidity and mortality in Western societies. In the UK, asthma costs >£2 billion/annum in healthcare expenditure, with the 10% of patients with severe asthma accounting for 50% of this resource^1^. Current approaches to treat asthma are clearly poorly effective for a sizeable proportion of patients which places a substantial social and economic burden. Additional new therapeutic approaches are therefore needed if this burden is to be reduced.

The main anti-inflammatory therapies used in asthma management are the glucocorticoids. These have widespread effects which reduce the expression of pro-inflammatory molecules by a variety of structural and inflammatory cells in the airway. Glucocorticoids act via glucocorticoid nuclear receptors (GR) which act at several sites to reduce inflammation and remodeling^2,3^. Glucocorticoids switch on or transactivate some genes such as the β_2_-adrenoceptor whilst switching off or transrepressing others (such as chemokine genes) by complex interactions involving transcription factors and their coactivators. Post-transcriptional modification of protein production is also important^4^. The fact that activated GR can translocate to the nucleus and modify the transcription of a wide range of inflammatory genes is one of the major reasons why glucocorticoids are so effective in asthma. Glucocorticoid receptors are not the only nuclear receptors found in mammalian cells however and other families of nuclear receptor are potential targets for new therapeutic approaches in inflammatory diseases such as asthma.

Peroxisome proliferator-activated receptors (PPARs) belong to a subfamily of the nuclear receptor superfamily and are important transcription factors that modulate inflammatory responses^5^. PPARs comprise three isoforms α, δ and γ and are activated by a heterogeneous group of structurally dissimilar endogenous and synthetic agonists. The prostaglandin D_2_ metabolite 15-deoxy-
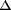
^12,14^PGJ_2_ (15d-PGJ_2_) is a direct-binding natural ligand for PPAR-γ. With the finding that PPAR-γ is important in cell metabolism a number of pharmaceutical companies have developed PPAR-γ agonists such as rosiglitazone and pioglitazone for the treatment of diabetes mellitus which act to reduce peripheral insulin resistance^6^.

PPAR-γ activation has been known to down regulate the synthesis and release of immune modulating cytokines from various cell types^6^. There is mounting evidence from studies in human cell systems and mouse asthma models to suggest that that activation of PPAR-γ has potent anti-inflammatory and immunomodulatory effects which may make this class of drugs candidates for the treatment of asthma^7-16^. Inflammatory cells such as eosinophils, mast cells, T lymphocytes and neutrophils are recruited into the airways in asthma by a network of chemokines. Stimulation of the PPAR-γ ligand was found to significantly inhibit production of the Th2 type cytokines and downregulate eosinophil functions^17,18^. Additionally treatment with synthetic PPAR-γ ligand can reduce lung inflammation and INF-γ, IL-4 and IL-2 production in experimental allergic asthma^19^. In subjects with asthma, PPAR-γ expression is know to be associated with airway inflammatory and remodelling responses^20^.

We have been studying the effect of PPAR-γ agonists on release of chemokines from cultured human airway smooth muscle cells, which we and others have shown are an important source of chemokines and inflammatory mediators in asthma^21-29^. We have shown that the endogenous PPAR-γ agonist 15d-PGJ2 and the synthetic PPAR-γ agonist troglitazone, but not the synthetic PPAR-α agonist WY-14643, inhibit TNFα-induced production of eotaxin and MCP-1, chemokines that selectively recruit eosinophils monocytes and T lymphocytes, but have no effect on the production of IL-8, the neutrophil chemoattractant. The findings suggest that both endogenous and synthetic PPAR-γ agonists act similarly to selectively modulate chemokines which are involved in allergic inflammation (Figure 1)^9^. Experiments transfecting cells with a wild type eotaxin promoter construct linked to firefly luciferase showed that this effect was on eotaxin transcription and was PPAR-γ mediated as overexpression of PPAR-γ enhanced the inhibitory effect (Figure 2). Furthermore we have also shown that PPAR-γ agonists increase release of PGE2, one of the most important endogenous bronchoprotective factors, through the induction of the enzyme cyclooxygenase-2^26^. Collectively these studies demonstrate a potentially useful anti-inflammatory profile of PPAR-γ agonists in a key airway structural cell.

Figure 1. The endogenous PPAR-γ agonist 15d-PGJ_2_ and the synthetic PPAR-γ agonist troglitazone (TRO), but not the synthetic PPAR-α agonist WY-14643, inhibit TNFα-induced production of eotaxin (left panel) and MCP-1 (middle panel), but not IL-8 (right panel), in human airway smooth muscle cells.

Figure 2. The endogenous PPAR-γ agonist 15d-PGJ_2_ and the synthetic PPAR-γ agonist troglitazone (TRO), but not the synthetic PPAR-α agonist WY-14643, inhibit TNFα-induced eotaxin promoter activity (left panel) and PPAR-γ overexpression enhances the inhibitory effect of 15d-PGJ_2_ (right panel) in human airway smooth muscle cells.

The anti-inflammatory effects of PPAR-γ agonists are not limited to airway structural cells, however. PPAR-γ agonists inhibit inflammatory cytokine release from monocytes, inhibit dendritic cell activation and induce apoptosis of T-lymphocytes^30-32^. Studies from mouse models of airway inflammation and asthma also support the hypothesis that activation of PPAR-γ has potent anti-inflammatory effects. PPAR-γ agonists reduced airway hyperresponsiveness, eosinophilic inflammation, and collagen deposition after allergen challenge in mice^10,12-15^.

A further feature of our studies is that as well as having anti-inflammatory effects on their own, PPAR-γ agonists potentiated the inhibitory effects of glucocorticoids^9^ suggesting that their may be benefits from using the drugs in combination with steroids. Figure 3 (next page) shows that the endogenous PPAR-γ agonist, 15d-PGJ_2_ had additive effects when given with the glucocorticoid, fluticasone in inhibiting TNFα-induced eotaxin release from human airways smooth muscle cells. We do not intend to study the interaction with glucocorticoids in the present study but this would provide a focus for follow on studies.

Figure 3. The endogenous PPAR-γ agonist, 15d-PGJ_2_ and the glucocorticoid fluticasone additively inhibit TNFα-induced eotaxin production in human airways smooth muscle cells.

Collectively these studies provide compelling evidence to suggest that PPAR-γ agonists may have beneficial effects in airways diseases such as asthma when given on their own or in combination with glucocorticoids. We therefore propose to explore the potential therapeutic effects of stimulating the PPAR-γ receptor by using the PPAR-γ agonist pioglitazone (licensed for use in diabetes) to treat people with asthma and to explore in detail the effects of PPAR-γ receptor stimulation on FEV_1_, sputum markers of airway inflammation and document any adverse effects.

## DETAILS OF INVESTIGATIONAL MEDICINAL PRODUCT(S)

### Description

30mg and 45mg over-encapsulated pioglitazone tablets. Pioglitazone tablets will be sourced from Takeda UK Ltd; please see separate Summary of Product Characteristics document (SmPC) that accompanies the protocol. Catalent Pharma Solutions will over-encapsulate the Pioglitazone tablets and perform dissolution testing and EU QP release.

### Packaging and labelling

The drug will be packaged and labelled for individual study participants by an independent pharmacist. Standard pharmacy supplies will be used.

###

### Storage, dispensing and return

IMP will be stored in a securely locked cupboard in the pharmacy at City Hospital, Nottingham only accessible to authorised pharmacy personnel. Supplies will be kept separately to other pharmacy supplies and labelled accordingly. When dispensed to participants, IMPs will be stored in their study packaging. Surplus or unused stock will be returned to the pharmacy for disposal.

### Placebo

The placebo will consist of a lactose filled capsule (lactose monohydrate is one of the excipients in Actos) and will be labelled, distributed and stored in the same way as for the IMP. The placebo capsule will be manufactured by Catalent Pharma Solutions.

### Known Side Effects

Please see separate SmPC that accompanies this protocol.

# TRIAL / STUDY OBJECTIVES AND PURPOSE

## PURPOSE

The key objective is to determine whether stimulation of PPAR γ receptors (using Pioglitazone) is effective in patients with mild-to-moderate asthma. The study design will allow us to estimate the size of the effect, record potential adverse effects and explore the presumed mechanism of action in-vivo by measuring sputum markers of airway inflammation. Definite evidence of clinical efficacy and biological acivity in patients with asthma would justify exploring PPAR-γ stimulation as a novel therapeutic modality.

## PRIMARY OBJECTIVE

To determine the effect of 12 weeks’ treatment with pioglitazone on FEV_1_

(measured using a dry bellows spirometer, *Vitalograph Buckingham UK* as the larger of two values within 100mls)

## SECONDARY OBJECTIVES

To determine the effect of 12 weeks’ treatment with pioglitazone on change over 12 weeks in daily asthma symptoms, mean morning and evening PEF, Juniper asthma control questionnaire^33^ and asthma quality of life questionnaire scores^34^, exhaled nitric oxide level (NIOXX Flex, Aerocrine), bronchial hyper-responsiveness (PD_20_) to methacholine (Provojet, Ganshorn Medzin Elecronic GMBH), induced sputum cell counts and analysis, body mass index, random glucose, eosinophil count and adverse effects.

Detailed sputum analysis will be carried out as follows:

Our previous studies have suggested that the mechanism of action of PPAR-γ agonists in vitro involves effects on gene transcription. Gene transcription is a complex process involving binding of transcription factors to their recognition sequences on gene promoters. Access to these recognition sequences requires DNA to unravel and this process is controlled by covalent modification of core histone molecules around which DNA is wrapped. The most important covalent modification is acetylation of histone H4 which is regulated by two competing groups of enzymes, histone acetyl transferases (HATs) and histone deacetylases (HDACs). HATs increase transcription whereas HDACs reduce it. In our studies of eotaxin we found that acetylation of histone H4 was reduced by PPARγ agonists but we have not determined whether this is due to a reduced HAT or increased HDAC activity. To examine things further we will perform studies in cells obtained from induced sputum in subgroups of the participants to analyse 1) HDAC and HAT activities by HDAC Fluorescent Activity Assay Kit (BIOMOL) and HAT Assay Kit (Upstate Biotechnology). 2) PPAR-γ activation (nuclear translocation) by western blotting as described previously^9^. This will be conducted on a subset of samples from each group to confirm the effect of pioglitazone. We will also perform studies in supernatant obtained from induced sputum to analyse 3) the concentration of chemokines (eotaxin, MCP-1, IP-10) and growth factors (VEGF) by Luminex, (Luminex corporation, Austin TX); 4) effector mediators (cyst-leukotriens, histamine by ELISA as described previously ^9^ and eosinophilic cationic protein (Immunocap, Phadia UK). These analyses will be conducted with samples obtained from participants at baseline and week 12 (the end of each treatment). The results will be normalised against total cell numbers to allow comparison.

# TRIAL / STUDY DESIGN

## TRIAL / STUDY CONFIGURATION

Single centre randomised two parallel group, double blind placebo controlled trial. Please see separate schematic diagram of study design.

### Primary endpoint

FEV_1_ after 12 weeks treatment

### Secondary endpoint

Change over 12 weeks in daily asthma symptoms, mean morning and evening PEF, Juniper asthma control questionnaire and asthma quality of life questionnaire scores, exhaled nitric oxide level, bronchial hyper-responsiveness, induced sputum cell counts and analysis detailed above, adverse effects.

### Safety endpoints

Adverse effects (spontaneously reported, reported at clinic visits, reported on diary cards)

### Stopping rules and discontinuation

Since this is a pilot study we do not anticipate obtaining data before completion of the study that would lead to its discontinuation. For individual participants, discontinuation will be decided on an individual basis.

## RANDOMIZATION AND BLINDING

The treatment to which a patient is assigned will be determined by a computer generated pseudo-random code using random permuted blocks of randomly varying size, created by the Nottingham Clinical Trials Unit (CTU) in accordance with their standard operating procedure (SOP) and held on a secure server, employing the Stata add-in ralloc.

Participants will be allocated with equal probability to the two treatment arms, stratified according to whether they use a regular inhaled corticosteroid or short acting β_2_ agonist alone.

Access to the sequence will be confined to the CTU Data Manager and will be concealed until interventions have been assigned and recruitment, data collection, and all other trial-related assessments are complete.

On behalf of investigators an independent senior research pharmacist will access the treatment code for each participant by means of a password-protected remote, internet-based system developed and maintained by the Nottingham CTU.

Participants, those administering the interventions and those assessing the outcomes will be blinded to group assignment.

Participants will be enrolled by Dr Tim Harrison, Dr Kevin Mortimer, Dr Dominick Shaw, Professor Alan Knox or Dr John Anderson from asthma clinics in Nottingham. We will work with the Primary care research network (PCRN East Midlands and South Yorkshire) to coordinate recruitment from GP practices within Nottinghamshire, Bassetlaw, Derbyshire and Lincolnshire. Treatment will be provided by pharmacy according to the randomisation code.

Treatment allocation will be concealed until interventions are all assigned and recruitment, data collection and laboratory analyses are complete.

### Maintenance of randomisation codes and procedures for breaking code

Study medications (active and placebo) will be produced to MHRA standards by Catalent Pharma Solutions.

Investigators may identify the treatment for each participant in the event of a medical emergency when treatment is dependent on knowledge of the actual drug received. This will be by means of opening a sealed envelope containing the details of drug allocation to each participant.

If the treatment code for a participant is broken, an urgent meeting of the study personnel (Independent trials pharmacist, Dr T Harrison and Dr K Mortimer, Dr John Anderson or Professor Alan Knox). The date and reason(s) for breaking the code will be recorded. The participant will continue follow up unless consent is withdrawn.

When the un-blinding is part of managing an SAE, unblinding will be reported with the SAE, however, in cases where un-blinding was not associated with an SAE, such actions will be reported in a timely manner. The default will be to use the same timeline requirements for investigator reporting of SAEs (notification of Sponsor immediately as practicable by phone or fax, followed by a written narrative of the event within 48 hours).

At the end of the study, randomisation and code break envelopes will be returned to pharmacy for disposal.

Treatment allocations will be identified by revealing the randomisation code at the end of the study.

## TRIAL MANAGEMENT

This small pilot study will be managed by the investigators who will meet on a monthly basis to assess progress. The Trial management group (TMG) will include Dr T Harrison, Dr J Anderson, C Minter, A Samuels, Dr K Mortimer, Dr D Hodgson, Kiara Parker and Denise Barber.

## DURATION OF THE TRIAL / STUDY AND PARTICIPANT INVOLVEMENT

Planned start date 1^st^ January 2010

Anticipated duration 24 months

Duration of involvement per participant: 18 weeks (2 weeks run-in, 12 weeks intervention, observation visit at 16 weeks) Enrolment will begin in January 2010 and is anticipated to close in January 2011.

###

### End of the Trial

The end of the trial will be the last visit for treatment of the last recruited participant.

## SELECTION AND WITHDRAWAL OF PARTICIPANTS

### Recruitment

Participants will be recruited from a single centre, Nottingham University Hospitals Trust through respiratory out-patient clinics in Nottingham and from our register of volunteers with asthma (who have agreed to be contacted about research projects like this one) as well as from a number of primary care practices within the East Midlands.

The initial approach in secondary care will be from a member of the patient’s usual care team (which may include the investigator), and information about the trial will be on display in the relevant clinical areas. The investigator or their nominee, e.g. from the research team or a member of the participant’s usual care team, will inform the participant or their nominated representative (other individual or other body with appropriate jurisdiction), of all aspects pertaining to participation in the study. If needed, the usual hospital interpreter and translator services will be available to assist with discussion of the trial, the participant information sheets, and consent forms but the consent forms and information sheets will not be available printed in other languages. It will be explained to the potential participant that that entry into the trial is entirely voluntary and that their treatment and care will not be affected by their decision. It will also be explained that they can withdraw at any time but attempts will be made to avoid this occurrence. In the event of their withdrawal it will be explained that their data collected so far cannot be erased and we will seek consent to use the data in the final analyses where appropriate.

As we plan to recruit patients with mild-to-moderate asthma many of whom will be followed- up in primary care we also plan to recruit from a number of GP practices within the East Midlands & South Yorkshire Primary Care Research Network area (PCRN). The Primary Care Trusts we have identified include; Nottingham City PCT, Nottinghamshire County PCT, Bassetlaw PCT, Derbyshire County PCT and Lincolnshire Teaching PCT. We will work with the PCRN to identify a number of suitable GP surgeries, who will to search their databases to identify potential participants with mild-to-moderate asthma. Potential participants will be sent an invitation letter by post from their GP on practice headed note paper informing them of the study aims and level of participation required. It will be made clear in the invitation letter that involvement in the trial is entirely voluntary and their usual medical care will not be affected should they decide not to partake. The letter will provide a contact telephone number for interested patients’ or their nominated representative (other individual or other body with appropriate jurisdiction) to contact a member of the study team for more information. We will then be able to provide patient information leaflet and arrange a discussion of all aspects pertaining to participation in the study prior to screening visit in the same way as patients recruited from secondary care clinics.

We will also invite potential participants to find out information about the trial with an A4 sized Poster. This poster will be displayed on official notice boards within Nottingham University Hospitals Trust and in a reduced size format as a Newspaper advertisement in a variety of local Newspapers.

### Inclusion criteria

Aged 18-75 of either sex with a clinical diagnosis of asthma, FEV_1_ ≥ 60% predicted and an increase in FEV_1_ of greater than 12% following inhaled salbutamol 400μg or PEF variability >12% during run-in. Allowed medication: 0-800μg inhaled beclomethasone diproprionate or equivalent and as required short acting beta agonist.

### Exclusion criteria

Inability to produce sputum on induction, current smoking, >10 pack years smoking history, treatment with long acting beta-agonists, leukotriene antagonists or theophylline, liver or cardiovascular disease, oral steroid treatment or exacerbation within 6 weeks, females who are pregnant, lactating or not using adequate contraception, any contra-indication to pioglitazone (hypersensitivity to pioglitazone, cardiac failure, history of cardiac failure, hepatic impairment, diabetic ketoacidosis), oral or insulin treatment for diabetes, treatment with gemfibrozil or rifampicin or lactose intollerance.

### Expected duration of participant participation

Study participants will be participating in the study for 18 weeks.

### Removal of participants from therapy or assessments

Subjects may be withdrawn from the trial either at their own request or at the discretion of the Investigator. The subjects will be made aware that this will not affect their future care. Subjects will be made aware (via the information sheet and consent form) that should they withdraw the data collected to date cannot be erased and may still be used in the final analysis.

Data will be collected on reasons for and timing of discontinuation/withdrawal.

Enrolled participants who are not yet randomised will be replaced but participants who withdraw after randomisation will not be replaced.

Abrupt termination of study treatment is not anticipated to affect participant safety.

### Informed consent

All subjects will provide written informed consent. The Informed Consent Form will be signed and dated by the subject before they enter the trial. The Investigator will explain the details of the trial and provide a Participant Information Sheet, ensuring that the subject has sufficient time to consider participating or not. The Investigator will answer any questions that the subject has concerning study participation.

Informed consent will be collected from each subject before they undergo any interventions (including physical examination and history taking) related to the study. One copy of this will be kept by the subject, one will be kept by the Investigator, and a third will be retained in the patient’s hospital records.

Should there be any subsequent amendment to the final protocol, which might affect a subject’s participation in the trial, continuing consent will be obtained using an amended Consent form which will be signed by the subject.

## TRIAL / STUDY TREATMENT AND REGIMEN

*Pre-study consent visit*

Detailed verbal and written information will be provided in clinic or in the clinical sciences building and interested subjects will be asked to sign the consent form and return for a screening visit. Subjects will be asked to withhold inhaled β-agonists for 6 hours before each visit and caffeine-containing food and drink from the previous midnight.

*Screening visit*

At the screening visit, the inclusion and exclusion criteria will be checked as part of the medical history followed by clinical examination and measurement of body mass index. Venesection will be performed for full blood count, liver function test, random glucose and if the patient agreees a single additional EDTA sample will be taken (approximately 10mls) at the same time to prepare plasma to store in the laboratory for future testing in respiratory research studies. We will seek prior patient consent at the pre-study clinic visit as an ‘opt-in’ item on the patient consent form, for permission to save a sample of their plasma in the laboratory to permit future testing of novel biomarkers. This will be fully explained in the patient information leaflet and in person and collection would occur at the same time as venepuncture for safety monitoring. The blood sample will be taken by an experienced doctor or research nurse and cells from the blood will be discarded and no genetic testing will be performed on the stored plasma. Subsequently spirometry (Vitallograph; Buckingham, UK) with measurement of the FEV_1_ reversibility twenty minutes following administration of 400µcg of inhaled salbutamol via a spacer device (Volumatic^TM^, Allen and Hanburys UK) followed by sputum induction will be performed.

*Run-in period*

Subjects will enter a two week run in period to complete a diary of their morning and evening peak flow, asthma medication use, nocturnal awakenings and other asthma symptoms.

*Randomisation*

Subjects fulfilling all inclusion criteria with no exclusion criteria will enter the study and allocation to treatment order will be by a computer generated randomization list prepared by the Clinical Trials Unit, Nottingham University, with treatment allocated by an independent pharmacist.

*Treatment period*

Subjects will start treatment with 30mg pioglitazone or placebo daily, increasing to 45mg daily after 4 weeks for the remaining 8 weeks of treatment. Participants will attend for data collection at the same time of day +/-1.5 hours at 0, 4, 8, 12 and 16 weeks within 3 days of the protocol date.

*Data collection*

*Baseline*

The inclusion criteria will be checked again, 30 mg of trial medication or placebo dispensed as per randomisation code. Diary cards will be checked (symptoms and PEF), use of medication confirmed and adverse effects recorded. The Juniper asthma control and asthma quality of life questionaires completed in the validated paper format followed by measuement of body mass index and clinical examination. Subsequently exhaled nitric oxide, spirometry, methacholine challenge for PD_20_ and induced sputum will be measured.

*Week 1*

Patients will be contacted by the research team by telephone one week following randomisation to ensure there are no early problems with trial medication or diary card monitoring.

*Weeks 4 and 8*

Diary cards (symptoms and PEF) will be checked, use of medication confirmed, adverse effects recorded then 45mg of trial medication or placebo will be dispensed according to radomisation code at week 4 and week 8. Subsequently measuement of body mass index, clinical examination and spirometry performed. Venesection for liver function tests and random glucose will be performed at week 4.

*Week 12*

At week 12 the treatment phase of the study has ended. Diary cards (symptoms and PEF) will be checked, use of medication confirmed, adverse effects recorded, Juniper asthma control questionnaire and asthma quality of life questionnaire completed. Subsequently clinical examiation, body mass index, measurement of exhaled nitric oxide , spirometry, methacholine challenge, induced sputum and venesection for full blood count, liver function test and glucose in that order.

*Week 16 (Observation)*

Diary cards (symptoms and PEF) will be checked, adverse effects recorded. Subsequently the Juniper asthma control questionnaire, asthma quality of life questionnaire completed and spirometry performed.

### Compliance

Compliance will be assessed by review of diary cards and counting of returned medication.

### Accountability for drugs & placebos

Dispensing will occur from pharmacy and dates and quantities recorded in each participant’s CRF. Dates and quantities of returned medication will also be recorded and confirmation of destruction or return of partially used/unused supplies recorded in pharmacy and CRF.

### Management of study drug overdose

Specific antidotes are not available. General appropriate supportive measures should be used with discussion with the local poisons advice centre if appropriate.

### Criteria for terminating trial

Since this is a pilot study we do not anticipate obtaining data before completion of the study that would lead to its discontinuation. For individual participants, discontinuation will be decided on an individual basis.

#

# STATISTICS

### Methods

The findings will be evaluated by Dr Harrison, Dr Mortimer, Dr Anderson, Dr Pang, Professor Knox and Dr Silcocks.

The analysis of the Primary response variable (FEV_1_ at 12 weeks) will be performed using a General Linear Model incorporating terms for baseline value, treatment arm, stratum, and baseline characteristics which are thought *a priori* to strongly predict outcome.

Secondary response variables will be treated similarly, after transformation to approximate Normality as required (as, for example with PD20, Percentage eosinophil count and exhaled NO concentrations). Mean morning and evening PEFR and the percentage of days and nights free of symptoms and relief inhaler use will be calculated for the 14 days prior to each visit and analysed as above. All analyses will be performed using Stata v10.

No interim or sub-group analyses are planned.

Full details will be given in a separate Statistical Analysis Plan to be approved before data lock.

### Sample size and justification

Assuming the SD of differences in FEV_1_ over 8 weeks is 250^35^ a study with 88 participants will allow us to detect a difference between treatment arms in the mean 12 week FEV_1_ of 150 ml with 80 % power at a 5% two-sided significance level. (Stata v10). In practice we aim to recruit 100 subjects to allow for drop-outs.

### Assessment of efficacy

Primary and secondary efficacy endpoints detailed above.

### Assessment of safety

Safety endpoints detailed above.

### Procedures for missing, unused and spurious data

Missing, unused and spurious data will be reported as such in the ensuing publication.

### Definition of populations analysed

Safety set: All randomised participants who receive at least one dose of the study drug.

Full Analysis set: All randomised participants, who take at least one dose of study medication and for whom at least one post-baseline assessment of the primary endpoint is available.

Per protocol set: All participants in the Full Analysis set who are deemed to have no major protocol violations that could interfere with the objectives of the study.

Efficacy will be assessed on both the full analysis set and the per protocol set. Safety summarises will be performed on the safety set.

# ADVERSE EVENTS

### Definitions

An adverse event is any unfavourable and unintended sign, symptom, syndrome or illness that develops or worsens during the period of observation in the study.

An AE does include a / an:

1. exacerbation of a pre-existing illness.

2. increase in frequency or intensity of a pre-existing episodic event or condition.

3. condition detected or diagnosed after medicinal product administration even though it may have been present prior to the start of the study.

4. continuous persistent disease or symptoms present at baseline that worsen following the start of the study.

An AE does not include a / an:

1. medical or surgical procedure (e.g., surgery, endoscopy, tooth extraction, transfusion); but the condition that lead to the procedure is an AE.

2. pre-existing disease or conditions present or detected at the start of the study that did not worsen.

3. situations where an untoward medical occurrence has not occurred (e.g., hospitalisations for cosmetic elective surgery, social and / or convenience admissions).

4. disease or disorder being studied or sign or symptom associated with the disease or disorder unless more severe than expected for the participant’s condition.

5. overdose of concurrent medication without any signs or symptoms.

A Serious Adverse Event (SAE) is any adverse event occurring following study mandated procedures, having received the IMP or placebo that results in any of the following outcomes:

1. Death

2. A life-threatening adverse event

3. Inpatient hospitalisation or prolongation of existing hospitalisation

4. A disability / incapacity

5. A congenital anomaly in the offspring of a participant

Important medical events that may not result in death, be life-threatening, or require hospitalisation may be considered a serious adverse event when, based upon appropriate medical judgment, they may jeopardize the patient or participant and may require medical or surgical intervention to prevent one of the outcomes listed in this definition

All adverse events will be assessed for seriousness, expectedness and causality:

A distinction is drawn between serious and severe AEs. Severity is a measure of intensity whereas seriousness is defined using the criteria above. Hence, a severe AE need not necessarily be serious.

### Causality

**Not related or improbable**: a clinical event including laboratory test abnormality with temporal relationship to trial treatment administration which makes a causal relationship incompatible or for which other drugs, chemicals or disease provide a plausible explanation**.** This will be counted as “unrelated” for notification purposes.

**Possible**: a clinical event, including laboratory test abnormality, with temporal relationship to trial treatment administration which makes a causal relationship a reasonable possibility, but which could also be explained by other drugs, chemicals or concurrent disease. This will be counted as “related” for notification purposes.

**Probable**: a clinical event, including laboratory test abnormality, with temporal relationship to trial treatment administration which makes a causal relationship a reasonable possibility, and is unlikely to be due to other drugs, chemicals or concurrent disease. This will be counted as “related” for notification purposes.

**Definite**: a clinical event, including laboratory test abnormality, with temporal relationship to trial treatment administration which makes a causal relationship a reasonable possibility, and which can definitely not be attributed to other causes. This will be counted as “related” for notification purposes.

An AE whose causal relationship to the study IMP is assessed by the Chief Investigator as “possible”, “probable”, or “definite” is an Adverse Drug Reaction.

With regard to the criteria above, medical and scientific judgment shall be used in deciding whether prompt reporting is appropriate in that situation.

### Reporting of adverse events

Participants will be asked to contact the study site immediately in the event of any serious adverse event. All adverse events will be recorded and closely monitored until resolution, stabilisation, or until it has been shown that the study medication or treatment is not the cause. The Chief Investigator shall be informed immediately of any serious adverse events and shall determine seriousness and causality in conjunction with any treating medical practitioners.

In the event of a pregnancy occurring in a trial participant or the partner of a trial participant monitoring shall occur during the pregnancy and after delivery to ascertain any trial related adverse events in the mother or the offspring. Where it is the partner of a trial participant consent will be obtained for this observation from both the partner and her medical practitioner.

All serious adverse events will be recorded and reported to the MHRA and REC as part of the annual reports. SUSARs will be reported within the statutory timeframes to the MHRA and REC as stated below. The Chief Investigator shall be responsible for all adverse event reporting.

## SUSARs

**A serious adverse event that is either sudden in its onset, unexpected in its severity and seriousness or not a known side effect of the IMP *and* related or suspected to be related to the IMP is classed as Suspected Unexpected Serious Adverse Reaction and requires expedited reporting as per the clinical trials regulations.**

**All serious adverse events that fall or are suspected to fall within these criteria shall be treated as a SUSAR until deemed otherwise.**

**The event shall be reported immediately of knowledge of its occurrence to the Chief Investigator.**

**The Chief Investigator will:**

- Assess the event for seriousness, expectedness and relatedness to the study IMP
- Take appropriate medical action, which may include halting the trial and inform the Sponsor of such action
- If the event is deemed a SUSAR, shall, within seven days, complete the CIOMS form and send to the MHRA.
- Shall inform the REC using the reporting form found on the NRES web page within 7 days of knowledge of the event
- Shall, within a further eight days send any follow-up information and reports to the MHRA and REC.
- Make any amendments as required to the study protocol and inform the ethics and regulatory authorities as required

### Participant removal from the study due to adverse events

Any participant who experiences an adverse event may be withdrawn from the study at the discretion of the Investigator.

# ETHICAL AND REGULATORY ASPECTS

## ETHICS COMMITTEE AND REGULATORY APPROVALS

The trial will not be initiated before the protocol, informed consent forms and participant and GP information sheets have received approval / favourable opinion from the Medicines and Healthcare products Regulatory Agency (MHRA), Research Ethics Committee (REC), and the respective National Health Service (NHS) Research & Development (R&D) department. Should a protocol amendment be made that requires REC approval, the changes in the protocol will not be instituted until the amendment and revised informed consent forms and participant and GP information sheets (if appropriate) have been reviewed and received approval / favourable opinion from the REC and R&D departments. A protocol amendment intended to eliminate an apparent immediate hazard to participants may be implemented immediately providing that the MHRA, R&D and REC are notified as soon as possible and an approval is requested. Minor protocol amendments only for logistical or administrative changes may be implemented immediately; and the REC will be informed.

The trial will be conducted in accordance with the ethical principles that have their origin in the Declaration of Helsinki, 1996; the principles of Good Clinical Practice, in accordance with the Medicines for Human Use Regulations, Statutory Instrument 2004, 1031 and its subsequent amendments and the Department of Health Research Governance Framework for Health and Social care, 2005.

## INFORMED CONSENT AND PARTICIPANT INFORMATION

The process for obtaining participant informed consent or assent and parent / guardian informed consent will be in accordance with the REC guidance, and Good Clinical Practice (GCP) and any other regulatory requirements that might be introduced. The investigator or their nominee and the participant or other legally authorised representative shall both sign and date the Informed Consent Form before the person can participate in the study.

The participant will receive a copy of the signed and dated forms and the original will be retained in the Trial Master File. A second copy will be filed in the participant’s medical notes and a signed and dated note made in the notes that informed consent was obtained for the trial.

The decision regarding participation in the study is entirely voluntary. The investigator or their nominee shall emphasize to them that consent regarding study participation may be withdrawn at any time without penalty or affecting the quality or quantity of their future medical care, or loss of benefits to which the participant is otherwise entitled. No trial-specific interventions will be done before informed consent has been obtained.

The investigator will inform the participant of any relevant information that becomes available during the course of the study, and will discuss with them, whether they wish to continue with the study. If applicable they will be asked to sign revised consent forms.

If the Informed Consent Form is amended during the study, the investigator shall follow all applicable regulatory requirements pertaining to approval of the amended Informed Consent Form by the REC and use of the amended form (including for ongoing participants).

## RECORDS

### Drug accountability

Drug supplies will be kept in a secure, limited access storage area under the storage conditions specified by Pharmacy.

The investigator and the local site pharmacist shall maintain records of the study drug’s delivery to the pharmacy, an inventory at the site, the distribution to each participant, and the return to the pharmacy or alternative disposition of unused study drugs. These records will include dates, quantities received, batch / serial numbers, expiration dates, and the unique code numbers (patient trial number) assigned to the trial participant. Investigators and /or the local site pharmacists will maintain records that document adequately that the participants were provided with the correct study medication. These records will be part of each patient’s Case Report Form (CRF). All study medication packs and bottles received by the pharmacy shall be accounted for.

### Case Report Forms

Each participant will be assigned a trial identity code number, allocated at randomisation, for use on CRFs other trial documents and the electronic database. The documents and database will also use their initials (of first and last names separated by a hyphen or a middle name initial when available) and date of birth (dd/mm/yy). Data entry will occur directly into a web-based electronic CRF (eCRF) set-up and maintained by Nottingham Clinical Trials Unit. The eCRF includes data encryption for personal information as well as an audit trail to log all online data entry and any changes in the data fields.

CRFs will be treated as confidential documents and held securely in accordance with regulations. The investigator will make a separate confidential record of the participant’s name, date of birth, local hospital number or NHS number, and Participant Trial Number (the Trial Recruitment Log), to permit identification of all participants enrolled in the trial, in case additional follow-up is required.

CRFs shall be restricted to those personnel approved by the Chief or local Principal Investigator and recorded on the ‘Trial Delegation Log.’

All paper forms shall be filled in using black ballpoint pen. Errors shall be lined out but not obliterated by using correction fluid and the correction inserted, initialled and dated.

The Chief or local Principal Investigator shall sign a declaration ensuring accuracy of data recorded in the CRF.

### Source documents

Source documents shall be filed at the investigator’s site and may include but are not limited to, consent forms, current medical records, laboratory results and pharmacy records. A CRF may also completely serve as its own source data. Only trial staff as listed on the Delegation Log shall have access to trial documentation other than the regulatory requirements listed below.

### Direct access to source data / documents

The CRF and all source documents, including progress notes and copies of laboratory and medical test results shall made be available at all times for review by the Chief Investigator, Sponsor’s designee and inspection by relevant regulatory authorities (e.g., MHRA, EMEA).

## DATA PROTECTION

All trial staff and investigators will endeavour to protect the rights of the trial’s participants to privacy and informed consent, and will adhere to the Data Protection Act, 1998. The CRF will only collect the minimum required information for the purposes of the trial. CRFs will be held securely, in a locked room, or locked cupboard or cabinet. Access to the information will be limited to the trial staff and investigators and relevant regulatory authorities (see above). Computer held data including the trial database will be held securely and password protected. All data will be stored on a secure dedicated web server. Access will be restricted by user identifiers and passwords (encrypted using a one way encryption method).

Information about the trial in the participant’s medical records / hospital notes will be treated confidentially in the same way as all other confidential medical information.

Electronic data will be backed up every 24 hours to both local and remote media in encrypted format.

# QUALITY ASSURANCE & AUDIT

## INSURANCE AND INDEMNITY

Insurance and indemnity for trial participants and trial staff is covered within the NHS Indemnity Arrangements for clinical negligence claims in the NHS, issued under cover of HSG (96)48. There are no special compensation arrangements, but trial participants may have recourse through the NHS complaints procedures.

The University of Nottingham has taken out an insurance policy to provide indemnity in the event of a successful litigious claim for proven non-negligent harm.

## TRIAL CONDUCT

Trial conduct will be subject to systems audit of the Trial Master File for inclusion of essential documents; permissions to conduct the trial; Trial Delegation Log; CVs of trial staff and training received; local document control procedures; consent procedures and recruitment logs; adherence to procedures defined in the protocol (e.g. inclusion / exclusion criteria, correct randomisation, timeliness of visits); adverse event recording and reporting; drug accountability, pharmacy records and equipment calibration logs.

The Trial Coordinator, or where required, a nominated designee of the Sponsor, shall carry out a site systems audit at least yearly and an audit report shall be made to the Trial Steering Committee.

## TRIAL DATA

Monitoring of trial data shall include confirmation of informed consent; source data verification; data storage and data transfer procedures; local quality control checks and procedures, back-up and disaster recovery of any local databases and validation of data manipulation. The Trial Coordinator, or where required, a nominated designee of the Sponsor, shall carry out monitoring of trial data as an ongoing activity.

Entries on CRFs will be verified by inspection against the source data. A sample of CRFs (10%) will be checked on a regular basis for verification of all entries made. In addition the subsequent capture of the data on the trial database will be checked. Where corrections are required these will carry a full audit trail and justification.

A Trial Steering committee will also convene to include, Professor A Knox, Dr T Harrison, Dr J Anderson, Dr K Mortimer, an independent chair (Professor Anne Tattersfield) and two other independent members.

An independent Data Monitoring and Ethics (DMEC) will be established which will report to the TSC. The DMEC will be chaired by Dr Dave Singh. The DMEC will have access to all available study data with the option to unblind the data if considered necessary for the safety of the participants.

Trial data and evidence of monitoring and systems audits will be made available for inspection by the regulatory authority as required.

## RECORD RETENTION AND ARCHIVING

In compliance with the ICH/GCP guidelines, regulations and in accordance with the University of Nottingham Research Code of Conduct, the Chief or local Principal Investigator will maintain all records and documents regarding the conduct of the study. These will be retained for at least 7 years or for longer if required. If the responsible investigator is no longer able to maintain the study records, a second person will be nominated to take over this responsibility.

The Trial Master File and trial documents held by the Chief Investigator on behalf of the Sponsor shall be finally archived at secure archive facilities at the University of Nottingham. This archive shall include all trial databases and associated meta-data encryption codes.

## DISCONTINUATION OF THE TRIAL BY THE SPONSOR

The Sponsor reserves the right to discontinue this trial at any time for failure to meet expected enrolment goals, for safety or any other administrative reasons. The Sponsor shall take advice from the Trial Steering Committee and Data Monitoring Committee as appropriate in making this decision.

## STATEMENT OF CONFIDENTIALITY

Individual participant medical information obtained as a result of this study are considered confidential and disclosure to third parties is prohibited with the exceptions noted above.

Participant confidentiality will be further ensured by utilising identification code numbers to correspond to treatment data in the computer files.

Such medical information may be given to the participant’s medical team and all appropriate medical personnel responsible for the participant’s welfare.

Data generated as a result of this trial will be available for inspection on request by the participating physicians, the University of Nottingham representatives, the REC, local R&D Departments and the regulatory authorities.

# PUBLICATION AND DISSEMINATION POLICY

We intend to submit the study results for publication. Participants will not be identified in any publications.

# USER AND PUBLIC INVOLVEMENT

We hold regular meetings for service users which are an opportunity to present the findings of recently completed research, discuss ongoing work and invite participation of service users in development of future work such as the current study. The participant information sheet for current study was prepared with input from one of our service users. In addition one service user will be appointed to as one of the two independent members of the Trial Steering Committee (TSC).

# STUDY FINANCES

### Funding source

This study is funded by the Nottingham University Biomedical Research Unit and a successful application has been submitted to the Efficacy and Mechanism Evaluation programme for funding.

###

### Participant stipends and payments

Participants will be given an inconvenience allowance of £25. Travel expenses will be offered for any hospital visits in excess of usual care.

#

# REFERENCES

1. National Asthma Audit 1999/2000; 1999. Direct Publishing Solutions Ltd.

2: Knox AJ, Pang L. Glucocorticoid and beta-adrenoceptor agonist interactions in asthma. Lancet. 2002 Oct 26;360(9342):1265-6.

3: Knox AJ. The scientific rationale of combining inhaled glucocorticoids and long acting beta 2 adrenoceptor agonists. Curr Pharm Des. 2002;8(20):1863-9.

4: Saklatvala J, Dean J, Clark A. Control of the expression of inflammatory response genes.

Biochem Soc Symp. 2003;(70):95-106.

5: Kersten S, Desvergne B, Wahli W. Roles of PPARs in health and disease. Nature. 2000 May 25;405(6785):421-4.

6: Perfetti R, D'Amico E. Rational Drug Design and PPAR Agonists. Curr Diab Rep. 2005 Oct;5(5):340-5. Jiang C, Ting AT and Seed B, PPAR-gamma agonists inhibit production of monocyte inflammatory cytokines, *Nature* **391** (6662) (1998), pp. 82–86.

7: Kim SR, Lee KS, Park HS, Park SJ, Min KH, Jin SM, Lee YC. Involvement of IL-10 in PPAR{gamma}-mediated anti-inflammatory response in asthma. Mol Pharmacol. 2005 Sep 8; [Epub ahead of print]

8: Lee KS, Park SJ, Hwang PH, Yi HK, Song CH, Chai OH, Kim JS, Lee MK, Lee YC. PPAR-gamma modulates allergic inflammation through up-regulation of PTEN. FASEB J. 2005 Jun;19(8):1033-5.

9: Nie M, Corbett L, Knox AJ, Pang L. Differential regulation of chemokine expression by peroxisome proliferator-activated receptor gamma agonists: interactions with glucocorticoids and beta2-agonists. J Biol Chem. 2005 Jan 28;280(4):2550-61.

10: Honda K, Marquillies P, Capron M, Dombrowicz D. Peroxisome proliferator-activated receptor gamma is expressed in airways and inhibits features of airway remodeling in a mouse asthma model. J Allergy Clin Immunol. 2004 May;113(5):882-8.

11: Ward JE, Gould H, Harris T, Bonacci JV, Stewart AG. PPAR gamma ligands, 15-deoxy-delta12,14-prostaglandin J2 and rosiglitazone regulate human cultured airway smooth muscle proliferation through different mechanisms. Br J Pharmacol. 2004 Feb;141(3):517-25.

12: Hammad H, de Heer HJ, Soullie T, Angeli V, Trottein F, Hoogsteden HC, Lambrecht BN. Activation of peroxisome proliferator-activated receptor-gamma in dendritic cells inhibits the development of eosinophilic airway inflammation in a mouse model of asthma. Am J Pathol. 2004 Jan;164(1):263-71.

13: Mueller C, Weaver V, Vanden Heuvel JP, August A, Cantorna MT. Peroxisome proliferator-activated receptor gamma ligands attenuate immunological symptoms of experimental allergic asthma. Arch Biochem Biophys. 2003 Oct 15;418(2):186-96.

14: Woerly G, Honda K, Loyens M, Papin JP, Auwerx J, Staels B, Capron M, Dombrowicz D. Peroxisome proliferator-activated receptors alpha and gamma down-regulat allergic inflammation and eosinophil activation. J Exp Med. 2003 Aug 4;198(3):411-21.

15: Trifilieff A, Bench A, Hanley M, Bayley D, Campbell E, Whittaker P. PPAR-alpha and -gamma but not -delta agonists inhibit airway inflammation in a murine model of asthma: in vitro evidence for an NF-kappaB-independent effect. Br J Pharmacol. 2003 May;139(1):163-71.

16: Benayoun L, Letuve S, Druilhe A, Boczkowski J, Dombret MC, Mechighel P,

Megret J, Leseche G, Aubier M, Pretolani M. Regulation of peroxisome proliferator-activated receptor gamma expression in human asthmatic airways: relationship with proliferation, apoptosis, and airway remodeling. Am J Respir Crit Care Med. 2001;164:1487-94.

17: G. Woerly, K. Honda and M. Loyens *et al.*, Peroxisome proliferator-activated receptors α and γ down-regulate allergic inflammation and eosinophil activation, *J Exp Med* **198** (2003), pp. 411–421.

18: S. Ueki, Y. Matsuwaki and H. Kayaba *et al.*, Peroxisome proliferator-activated receptor gamma regulates eosinophil functions: a new therapeutic target for allergic airway inflammation, *Int Arch Allergy Immunol* **134** (Suppl. 1) (2004), pp. 30–36.

19: C. Mueller, V. Weaver and J.P. Vanden Heuvel *et al.*, Peroxisome proliferator-activated receptor gamma ligands attenuate immunological symptoms of experimental allergic asthma, *Arch Biochem Biophys* 418 (2) (2003), pp. 186–196.

20: L. Benayoun, S. Letuve and A. Druilhe *et al.*, Regulation of peroxisome proliferator-activated receptor gamma expression in human asthmatic airways: relationship with proliferation, apoptosis, and airway remodeling, *Am J Respir Crit Care Med* 164 (8 Pt. 1) (2001), pp. 1487–1494.

21: Nie M, Knox AJ, Pang L. beta2-Adrenoceptor agonists, like glucocorticoids, repress eotaxin gene transcription by selective inhibition of histone H4 acetylation. J Immunol. 2005 Jul 1;175(1):478-86.

22: Bradbury D, Clarke D, Seedhouse C, Corbett L, Stocks J, Knox A. Vascular endothelial growth factor induction by prostaglandin E2 in human airway smooth muscle cells is mediated by E prostanoid EP2/EP4 receptors and SP-1 transcription factor binding sites. J Biol Chem. 2005;280(34):29993-30000.

23: Elshaw SR, Henderson N, Knox AJ, Watson SA, Buttle DJ, Johnson SR. Matrix metalloproteinase expression and activity in human airway smooth muscle cells. Br J Pharmacol. 2004;142(8):1318-24.

24: Nie M, Pang L, Inoue H, Knox AJ. Transcriptional regulation of cyclooxygenase 2 by bradykinin and interleukin-1beta in human airway smooth muscle cells: involvement of different promoter elements, transcription factors, and histone h4 acetylation. Mol Cell Biol. 2003 Dec;23(24):9233-44.

25: Zhu YM, Bradbury DA, Pang L, Knox AJ. Transcriptional regulation of interleukin (IL)-8 by bradykinin in human airway smooth muscle cells involves prostanoid-dependent activation of AP-1 and nuclear factor (NF)-IL-6 and prostanoid-independent activation of NF-kappaB. J Biol Chem. 2003;278(31):29366-75.

26: Pang L, Nie M, Corbett L, Knox AJ. Cyclooxygenase-2 expression by nonsteroidal anti-inflammatory drugs in human airway smooth muscle cells: role of peroxisome proliferator-activated receptors. J Immunol. 2003 Jan 15;170(2):1043-51.

27: Pang L, Nie M, Corbett L, Donnelly R, Gray S, Knox AJ. Protein kinase C-epsilon mediates bradykinin-induced cyclooxygenase-2 expression in human airway smooth muscle cells. FASEB J. 2002 Sep;16(11):1435-7.

28: Knox AJ, Corbett L, Stocks J, Holland E, Zhu YM, Pang L. Human airway smooth muscle cells secrete vascular endothelial growth factor:up-regulation by bradykinin via a protein kinase C and prostanoid-dependent mechanism. FASEB J. 2001 Nov;15(13):2480-8.

29: Pang L, Knox AJ. Regulation of TNF-alpha-induced eotaxin release from cultured human airway smooth muscle cells by beta2-agonists and corticosteroids. FASEB J. 2001 Jan;15(1):261-269.

30: Appel S, Mirakaj V, Bringmann A, Weck MM, Grunebach F, Brossart P. PPAR-gamma agonists inhibit toll-like receptor mediated activation of dendritic cells via the MAP kinase and NF-kappaB pathways. Blood. 2005 Aug 16; [Epub ahead of print]

31: Cheron A, Peltier J, Perez J, Bellocq A, Fouqueray B, Baud L. 15-deoxy-Delta12,14-prostaglandin J2 inhibits glucocorticoid binding and signaling in macrophages through a peroxisome proliferator-activated receptor gamma-independent process. J Immunol. 2004 Jun 15;172(12):7677-83.

32: Tautenhahn A, Brune B, von Knethen A. Activation-induced PPARgamma expression sensitizes primary human T cells toward apoptosis. J Leukoc Biol. 2003 May;73(5):665-72.

33: Juniper EF, O'Byrne PM, Guyatt GH, Ferrie PJ, King DR. Development and validation of a questionnaire to measure asthma control Eur Respir J 1999;14:902-907.

### 34: **Juniper EF, Buist AX, Cox FM, Ferrie PJ, King DR.** [Validation of a Standardized Version of the Asthma Quality of Life Questionnaire](http://www.chestjournal.org/cgi/content/abstract/115/5/1265). Chest 1999;115:1265-1270.

35: [Fogarty A, Lewis SA, Scrivener SL, Antoniak M, Pacey S, Pringle M, Britton J.](http://www.ncbi.nlm.nih.gov/entrez/query.fcgi?cmd=Retrieve&db=pubmed&dopt=Abstract&list_uids=14519140&query_hl=2), Oral magnesium and vitamin C supplements in asthma: a parallel group randomized placebo-controlled trial. Clin Exp Allergy. 2003 Oct;33(10):1355-9.

36: Philips K, Oborne J, Lewis S, Harrison TW, Tattersfield AE. Time course of action of two inhaled corticosteroids, fluticasone propionate and budesonide. Thorax 2004;59:26-30.

**19. Flow diagram (please refer also to Appendix 1)**

Letters sent to potential participants identified from primary and secondary care lists (n ~ 200)

Suitability screening (n ~ 150)

Suitable participants enrolled (n ~ 120)

2 week run-in of PEF monitoring

Randomisation (n = 100)

One active capsule containing 30mg pioglitazone and lactose taken daily for four weeks (n = 50)

One placebo capsule containing lactose taken daily

for four weeks (n = 50)

One active capsule containing 45mg pioglitazone and lactose taken daily for eight weeks

One placebo capsule containing lactose taken daily for eight weeks

End of study. Primary and secondary outcome measures obtained after 12 weeks treatment

End of study. Primary and secondary outcome measures obtained after 12 weeks treatment

Final follow up visit 4 weeks after finishing treatment

Final follow up visit 4 weeks after finishing treatment

# SIGNATURE PAGES

Signatories to Protocol:

**Chief Investigator:** (name)__________________________________

Signature:__________________________________

Date: ___________

**Co- investigator**: (name) __________________________________

Signature:__________________________________

Date: ___________

**Trial Statistician**: (name)__________________________________

Signature:__________________________________

Date: ___________

**Trial Pharmacist**: (name)__________________________________

Signature:__________________________________

Date: ___________
